# The ribonucleoprotein hnRNPA1 mediates binding to RNA and DNA telomeric G-quadruplexes through an RGG-rich region

**DOI:** 10.1016/j.jbc.2025.108491

**Published:** 2025-04-08

**Authors:** Sangeetha Balasubramanian, Irawati Roy, Rajeswari Appadurai, Anand Srivastava

**Affiliations:** 1 Molecular Biophysics Unit, Indian Institute of Science, Bangalore, Karnataka, India; 2 Department of Biology, Indian Institute of Science Education and Research, Tirupati, Andhra Pradesh, India

## Abstract

hnRNPA1, a protein from the heterogeneous-nuclear ribonucleoprotein family, mediates cellular processes such as RNA metabolism and DNA telomere maintenance. Besides the folded RNA recognition motifs, hnRNPA1 has a ∼135 amino-acids long low-complexity domain (LCD) consisting of an RGG-rich region and a prion-like domain (PrLD). Biochemical data suggest that the RGG-rich region modulates the recognition of G-quadruplexes (GQs) in the telomeric repeats. Here, we utilize an in-house developed replica exchange technique (REHT) to generate the heterogeneous conformational ensemble of hnRNPA1-RGG and explore its functional significance in telomere maintenance. Single chain statistics and abundance of structural motifs, as well as consistency with experimentally reported structural data suggest faithful recapitulation of local interactions. We also introduce a protocol to generate functionally significant IDP-nucleic acid complex structures that corroborate well with the experimental knowledge of their binding. We find that RGG-box preferentially binds to the grooves and loops of GQs providing specificity towards certain GQ structures with its sequence and secondary structures. Turn-like structures expose Phe and promote stacking with the G-tetrads, while Tyr and Asn residues form essential hydrogen bonds and electrostatic interactions. Several of these residues were also identified as important by the earlier reported HSQC chemical shift data. Our binding and simulation studies also reveal that a minor population of the RGG-box can perturb telomeric GQs structure, which likely expedites the unfolding activities of hnRNPA1-UP1 at the telomeric end.

Biomolecular interactions between proteins and nucleic acids, particularly higher-order structures of nucleic acids like G-quadruplexes (GQ), have a critical role in gene regulation, telomere maintenance, genome stability, and other cellular processes (1). GQs are non-canonical super-secondary structures formed by G-rich repeats, (*e.g*. d(GGG[T/U][T/U]A)n in human telomeres) where guanines arrange in a square planar configuration to form G-quartets, stabilized by hydrogen bonds and cation coordination at the core. These quartets stack to form a stable three-layered GQ structure connected by loops. While the DNA GQs can exist in diverse topologies ranging from parallel or antiparallel strands of tetramolecular, bimolecular, and unimolecular forms based on the flanking sequence, cation type, and the interacting partners, the RNA GQs can adopt only a parallel structure coordinated by K_+_ ions (2).

Recognition of GQs by GQ-binding proteins tightly regulates downstream processes by stabilizing or destabilizing the GQ structures. For instance, the GQ binding proteins such as FUS, EWS, and Nucleolin are shown to stabilize the folded telomeric DNA and RNA GQs (3, 4). On the other hand, heterogenous nuclear ribonucleoprotein A1(hnRNPA1) and DEAD-box helicases unfold the GQs (5). The architecture of GQ binding proteins often consists of one or more structured domains such as RRM (RNA recognition motif) domains, OB-fold domains, and disordered RGG domains composed of Arg-Gly dipeptide or Arg-Gly-Gly tripeptide repeats. Besides the role of structured domains, the role of disordered RGG domains in GQ recognition has been widely recognized in recent times. GQ-binding proteins like helicases, hnRNP, FET family proteins, nucleolin, and FMRP among others, have one or more RRM domains and RGG-rich regions that function concertedly or individually to stabilize or destabilize the G-quadruplexes (5). Particularly, the RGG domains of FUS and EWS are shown to promote the folding of telomeric DNA and RNA GQs leading to telomere shortening and histone methylation at the telomeres (3, 4). In the case of nucleolin, both RRM and RGG domains are required to form a GQ in the c-myc promoter sequence and the RGG domain is important to stabilize this GQ (6). In the case of hnRNPA1, the RRM can function independently to unfold GQ (7–10). However, the presence of the RGG domain enhances the unfolding activity, which indicates the role of the RGG-box beyond that of a biomolecular recognition adaptor (11, 12).

The RGG-box of diverse GQ binding proteins is believed to exhibit distinct specificity based on its sequence composition. The RGG of translocated in liposarcoma (TLS) protein has been shown to bind both RNA and DNA GQ (13). However, mutating the Tyr residues in the RGG domain to Phe leads to specific binding with RNA GQ by recognizing the ribose 2’-OH exposed in the loops. Moreover, the Tyr-only RGG and Phe-only RGG constructs exhibit specific binding to telomeric DNA and RNA GQs, respectively (14). These studies also suggest that the specific recognition of GQs by RGG modulates histone methylation at the telomeres. With the objective to design the most consensus GQ-binding motif, Huang *et al*. (15) identified a 20-mer peptide (RGRGRGRGGGSGGSGG RGRG) with seven RG/RGG repeats having a high affinity to parallel GQs. Mutational study of this peptide clearly high-lights the role of amino acid composition and their position for GQ binding affinity. The role of Arg for GQ binding has been investigated previously and its importance is attributed to the multivalent behavior (16). Apart from their ability to form hydrogen bonds and π-stacking interactions with the nucleobases, the positively charged guanidine group is involved in electrostatic interactions with the negatively charged phosphate backbone of nucleic acids. Both Arg and Phe were shown as crucial for GQ binding due to their ability to form electrostatic as well as pi-stacking interactions. Gly residues in the RGG motif provide the necessary flexibility that allows adaptive binding to several GQ topologies. In general, GQ-binding proteins are rich in Glycine, Arginine, Aspartic acid, Asparagine, and Valine (17). Although some biochemical insights into RGG-GQ binding are available, the structural and mechanistic principles governing this interaction remain unclear due to its disordered nature. Recently, a similar motif, the F/YGG motif, has been identified as a nucleic-acid binding motif with an intrinsically disordered nature (18).

In this study, we aim to uncover high-resolution insights into the mechanism of RGG-mediated GQ binding and its impact on GQ structure, using the RGG domain of hnRNPA1 as an example. hnRNPA1 is a member of a complex and diverse group of proteins called hnRNPs that collectively play an important role in processing heterogeneous nuclear RNAs into mature mRNAs. This abundant nuclear protein modulates many RNA metabolism processes, including transcription (19–21), splicing (21–24), RNA stability, export (25–27), and translation (28–30). The role of hnRNPA1 is not just limited to mRNA bio-genesis but extends to other processes such as microRNA processing and telomere maintenance (31). hnRNPA1 promotes the capping of telomeres by direct binding with DNA-dependent protein kinase catalytic subunit, DNA-PKcs (32). Phosphorylation of hnRNP promotes Shelterin complex formation by recruiting POT1 (protection of telomeres 1) protein to telomeres which then associates with Shelterin complex-forming proteins (33). It also modulates the binding of telomerase regulatory proteins like RPA (replication protein A) (34). Apart from the proteins, hnRNP A1 can also bind to the single strand and G-quadruplex folds of both telomeric DNA and RNA (35). Telomerase activity requires access to single-stranded G-rich 3’-overhangs, however, these regions are known to form G-quadruplex structures. hnRNP A1 is shown to bind to the telomeric DNA GQs and unfold them for telomerase activity. Similarly, sequestration of hnRNP A1 by TERRA can lead to telomerase inhibition. The binding of hnRNP A1-TERRA has been shown to regulate TERRA-mediated inhibition of telomerase in a TERRA-concentration-dependent manner (11, 12, 36).

Structurally (Fig. 1>A), the N-terminus of hnRNPA1 contains two well-folded RNA recognition motifs (RRMs) connected by a small linker region. Jointly these two motifs constitute the unwinding protein 1 (UP1) (37), which mediates interactions with target RNAs. UP1 domain is shown to bind to and stabilize telomeric ssDNA and also unfold telomeric GQ (9, 31, 38–40). In addition, a flexible Glycine rich C-terminal region consisting of Arg-Gly-Gly tripeptide repeats (RGG), interspersed with aromatic (Phe, Tyr) residues to form RGG boxes (Fig. 1B), provide both protein and RNA binding capabilities to hnRNPA1 (37). Although the RGG domain of hnRNP A1 is called the “RGG-rich” region, this region contains five FG/FGG motifs and six Asparagines in the vicinity of Phe. Downstream from the RGG-box region, the C-terminal domain harbors a prion-like domain (PrLD) and a nuclear shuttling sequence called the M9 sequence (41). PrLD has been shown to induce protein-protein interaction, leading to stress granule assembly and pathological protein aggregation (41). The RGG and PrLD domains together constitute the disordered low-complexity domain (LCD) of hnRNPA1.

Several lines of evidence have demonstrated that both UP1 and RGG-box domains are involved in GQ unfolding activity at the telomeric end of DNA (11, 42). It has been shown previously that the GQ unfolding rate of the UP1 domain (k_obs_ = 0.63 ± 0.01) is lower than UP1+RGG (k_obs_ = 1.93 ± 0.01) (11). RGG-box might act as an adaptor for the UP1 by bringing it closer to the GQ, which leads to an efficient binding of UP1 followed by the unfolding of the GQ structure (11). Finally, UP1 can form a high-affinity complex with the single-stranded DNA and stabilizes the extended form. It is also possible that the RGG domain might induce perturbations in the GQ structure leading to faster and more efficient binding of UP1 domain, which has a preference for unfolded DNA/RNA. Despite tremendous strides made in the understanding of hnRNPA1 interactions with GQ using biochemical studies, high-resolution structural and dynamic insights into hnRNPA1-GQ interaction and telomere maintenance are still missing. In our work, we utilize the integrative molecular modeling approach that combines atomic-resolution molecular simulations with available ensemble-averaged data from NMR to faithfully reconstruct the atomic-resolution ensemble of hnRNPA1-RGG and use the structural data to investigate the functional role of RGG in telomere maintenance.

In this work, we have focused on and explored the structural heterogeneity and associated biological activities of hnRNPA1 with the help of our advanced sampling (43) and large-scale biomolecular simulations of hnRNPA1-RGG and its RNA/DNA GQ complexes. The article is organized as follows. In the Results and Discussion section following this Introduction section, we report the data from our extensive docking and simulations of RGG-region interacting with multiple RNA and DNA GQ topologies. Our simulations provide molecular-level insights into the reported role of the RGG-region in telomere maintenance. We discuss in detail how the RGG-region binds differently to telomeric DNA and telomeric RNA GQs and we also demonstrate how a very small population of hnRNPA1-RGG conformers are capable of perturbing the GQs. After the Results section, we briefly summarize our observations and findings in the Conclusions section, which is followed by the Materials and Methods where we provide details of all the methods that we have used. Additional information is included in the Supporting Information (SI) file. All our data including the simulated trajectories and input files for our simulations, all analyses-related data and codes are publicly available https://figshare.com/s/4eb0701bdde1af2c2711.

## Results and discussion

### Atomic resolution structural insights into the RGG conformation ensemble: conformations consistent with NMR data show a prevalence of β-motifs

**Figure 1:**
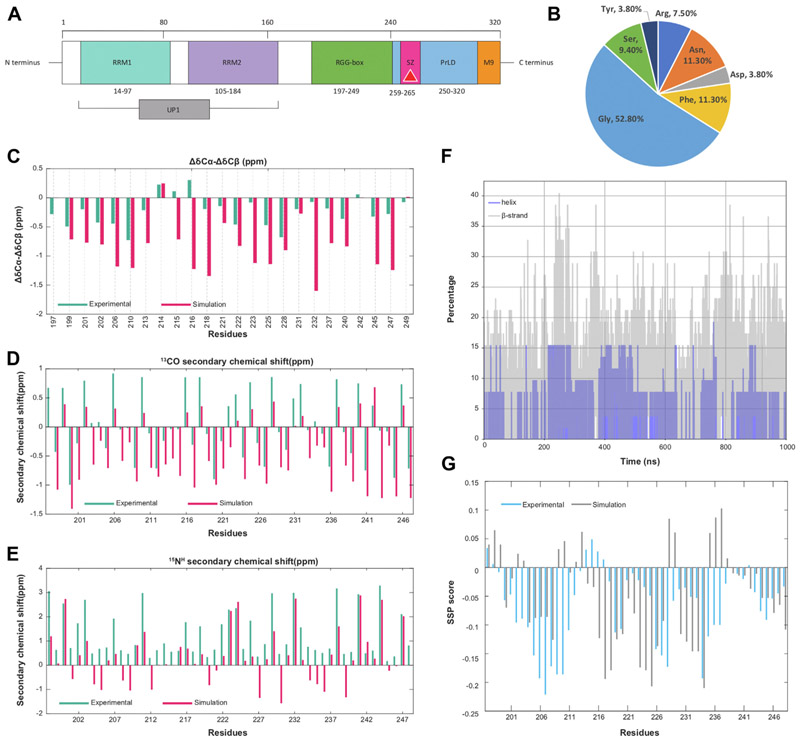
Sequence and structural insights into RGG-box domain ensemble. *A*, domain architecture of hnRNPA1. The *Teal* and *Purple* colored domains are the N terminal-ordered RNA recognition motifs. Together, these two domains are called UP1. The steric-zipper motif (259–265) is colored in *Magenta*. The *Green* and the *Blue* domains are intrinsically disordered RGG-box domain and PrLD, respectively. The *Orange* colored domain is the nuclear shuttling sequence M9. *B*, sequence composition of RGG-box motif. *C*–*E*, the ΔCα-ΔCβ, ^13^CO, and ^15^N^H^ secondary chemical shift plots for RGG, respectively. *Teal* is experimental data. *Dark pink* indicates simulation data. The ΔCα-ΔCβ chemical shifts are calculated and plotted for the residues containing Cβ atoms. *F*, comparison of α-helix (*gray*) and β-sheet (*blue*) content of RGG during the simulation. *G*, predicted secondary structure propensity scores from chemical shift data of experiments (*blue*) and simulation (*gray*) using the SSP algorithm. SSP score above 0 indicates helical propensity and below 0 indicates β-sheet propensity.

We obtained a heterogeneous ensemble of the RGG domain (197–249 amino acids) (Fig. 1A) to gain conformational insights by harnessing a Hamiltonian and temperature-scaled hybrid replica exchange technique called Replica Exchange with Hybrid Tempering (REHT) (43–45). Please refer to the Methods section for more details. We calculated the ΔCα-ΔCβ, ^13^CO, and ^15^N^H^ secondary chemical shifts using the conformation ensemble generated from our REHT simulation. The residue-wise comparison between the calculated data from the simulation and the experimentally available chemical shift data showed a considerable match (46) as shown in Figure 1, *C*–*E*. Upon performing a basic secondary structure analysis across the time evolution of our REHT ensemble, we observed a significant propensity to form transient secondary structures, preferentially β-sheet, and extended β-structures at different regions of RGG, as shown in Fig. S1 in SI. In a separate study, we have recently explored the abundance of transient and non-transient beta-sheet structures in the RGG domain of hnRNPA1 and low-complexity domains of other prion-like proteins (47). In general, we find that the overall percentage of β-structures in each frame is higher when compared to α-helices (Grey in Fig. 1*F*). To verify this, we also used the chemical shift data of the experiment and simulation ensemble to predict the secondary structure propensities per residue. The secondary structure prediction (SSP) (48) scores of both experimental and simulated data re-emphasize our secondary structure findings of the ensemble data by showing a higher β-sheet population compared to that of α-helix (Fig. 1*G*). Altogether, the secondary chemical shifts and secondary structure analysis suggest a heterogeneous ensemble with an overall inclination to form local contacts through transient secondary structure formation.

At this point, it may be pertinent to discuss the prevalent ambiguity regarding secondary structure prediction from chemical shift data using conformational ensembles generated from molecular simulations. First of all, at the experimental interpretation level itself, predicting the secondary structure of intrinsically disordered proteins (IDPs) from chemical shift data is often potentially ambiguous due to several key reasons. Some of these reasons are the lack of a well-defined secondary or tertiary structure under the physiological conditions of IDPs and IDRs, the chemical shift averaging over many conformations, the high dependence of the chemical shift of an IDP on its local environment, lack of suitable reference database and last but not the least, the weak secondary structure propensities. The weak and transient secondary structure propensities exhibited by IDPs often do not produce chemical shift deviations that are strong enough to be reliably detected and interpreted using conventional secondary structure prediction algorithms. Due to this specific reason, in our particular case of the RGG box, we have observed outcomes from SSP analysis that have to be interpreted in light of inherent ambiguity in making strong deductions from the experimental data itself. The use of ^13^C_α_, ^13^C_β_, and ^13^CO gave a higher percentage of α-helix propensity, whereas the use of ^13^C_α_, ^13^C_β_, and ^1^H_α_ produced a higher β-sheet propensity. Due to the effect of the weak secondary structure propensity, the reliability of the prediction gives way to obvious ambiguity. Nevertheless, in this work, we followed the recommended set of raw chemical shift data according to the SSP prescription of IDP (which are ^13^C_α_, ^13^C_β_, and ^1^H_α_) and were able to qualitatively match the result with the outcome of secondary structure calculation from our simulation data set using DSSP.

### Functional insights from RGG conformational ensemble: role of RGG box in telomere maintenance

**Figure 2:**
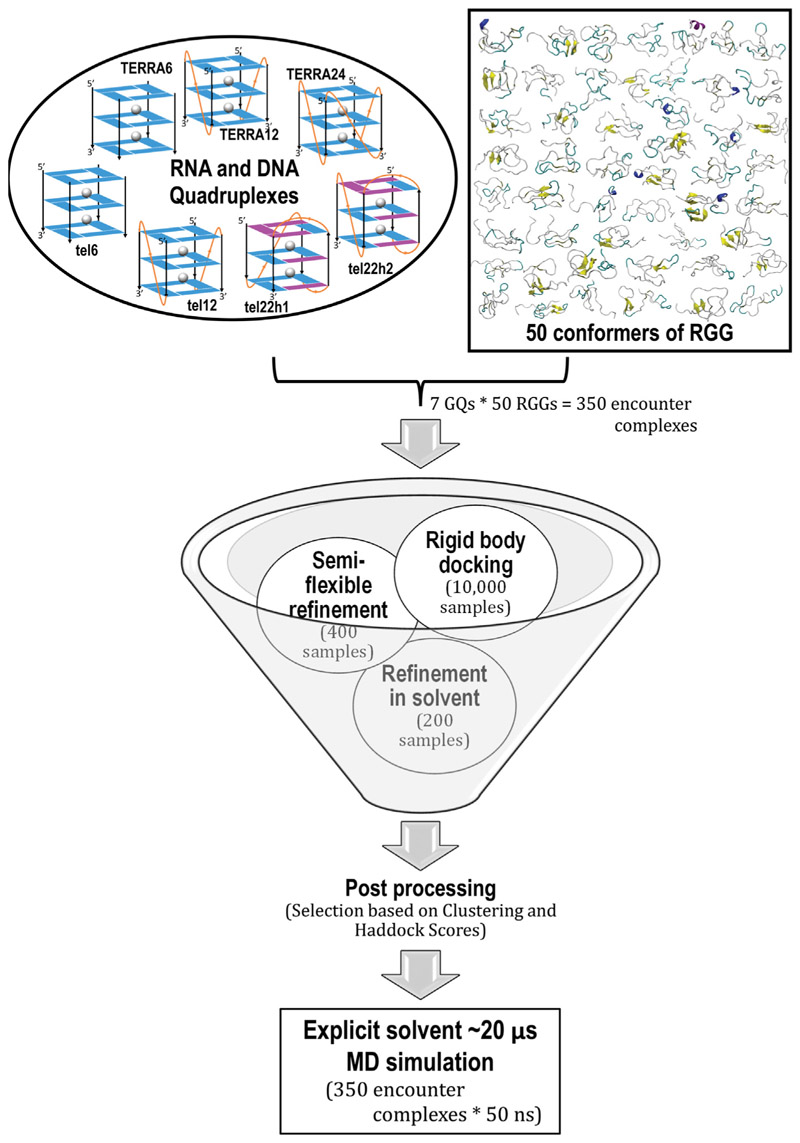
Flowchart of the protocol adopted to generate RGG-GQ complexes. Seven different telomeric DNA and RNA GQ structures chosen from the literature were docked with each of the 50 conformers of RGG obtained by t-SNE clustering of the generated ensemble. HADDOCK protocol with a center of mass distance criteria was used to reduce the sample space from 10,000 initial random geometries for each complex. The 350 (7 GQs with 50 RGGs) encounter complexes were further simulated for 50 ns each resulting in a 17.5 μs ensemble to explore the mode of binding, stability, and conformational dynamics of the RGG-GQ binding process.

In 2018, Ghosh and Singh compared the 15N-1H HSQC data of the UP1+RGG-box domain and the isolated RGG-box domain (11). A superposition of the NMR spectra of these two constructs showed that RGG occupies the same conformational space with or without the existence of UP1. This in turn sheds light on the modular nature of the UP1-RGG construct where the tethered RGG-box behaves as a structurally independent domain from the folded UP1 domain. This also opens up the possibility that the full-length LCD (RGG + PrLD) could be modular. Bioinformatics studies on sequence space have suggested distinct compositional boundaries within IDRs —revealing a possible non-random modular organization in the sequence space (49). Fortuitously, NMR studies from completely different groups have reported the HSQC spectra of the RGG domain (11) and the full-length LCD with the RGG domain and PrLD domain together as one chain (46). In order to explore the possibility that the full-length LCD of the hnRNPA1 could be modular, we superposed these HSQC spectra and it is clearly seen that the chemical shifts of RGG overlap in the two systems (Fig. S2A in SI). The NMR data clearly portrays the modularity of hnRNPA1 and signifies that the RGG domain, in spite of being disordered, can function independent of the other regions of hnRNPA1 such as UP1 and PrLD. This structural and functional modularity motivated us to dig deeper into the functionality of the RGG domain, in particular, our focus was to understand the functional relevance of the RGG box domain in telomere maintenance. hnRNPA1 expresses a concentration-dependent binding with telomeric RNA and DNA; in particular, it localizes at the GQ structural motif. Studies have hypothesized that the RGG-box binds to GQ, and recruits UP1 which in turn destabilizes the GQ (11, 12). We explore this hypothesis of RGG-GQ interactions, but the heterogeneity of the RGG ensemble complicates the selection of a reference RGG structure for the study. We used our recently developed machine learning-based clustering technique, which employs t-distributed stochastic neighbor embedding (t-SNE), to group the huge heterogeneous ensemble into 50 homogeneous clusters (50) (see Fig. S2B in SI). Our tSNE-based clustering algorithm for IDPs combines t-distributed stochastic neighbor embedding projection and kMeans clustering technique to group the heterogeneous data into homogenous subgroups. Through our Silhouette score, we can see that each cluster is evidently highly homogeneous, whereas the conformations between clusters are heterogeneous. The algorithm and parameters used to perform tSNE clustering is discussed in the Methods section. In a recent study, we have modeled the interaction of the RRM-RGG domains of FUS with a stem-loop RNA by all-atom molecular dynamics simulations (51). This study provided useful insights on the interaction of RGG with RNA and tSNE was used to characterize the conformational ensemble. We selected a central conformation from each cluster to represent the different states sampled by the RGG in solution and further used them to study the functional interactions. Being an IDP, RGG might interact with other partners *via* several mechanisms like “folding upon binding” or fuzzy interactions (52–54). In computational studies, first-pass docking methods generally provide insight into the formation of “encounter complexes”. To identify these encounter complexes and the probable basis for GQ recognition by RGG, we performed docking studies of RGG with telomeric DNA as well as RNA quadruplexes using HADDOCK. Further, the docked structures were then subjected to molecular simulations to capture the dynamics of the complex. A flowchart depicting the protocol adopted to generate the initial RGG-GQ encounter complexes is shown in Fig. 2.

### Encounter complexes of telomeric RNA GQ are “end stacking” and telomeric DNA GQ are “groove binding”

We docked each of the 50 conformations of RGGs with seven different unimolecular, bimolecular, and tetramolecular GQs of telomeric RNA (named TERRA6, TERRA12, and TERRA24, respectively) and DNA (named tel6, tel12, tel22h1, and tel22h2). The systems under consideration are listed in Table 1, and the HADDOCK scores for all complexes are plotted in Fig. S3 in SI. Preliminary visual inspection and analysis of three-dimensional occupancy maps (drawn on an isosurface with a 6 Å distance cutoff) reveal the most preferred binding sites on the 7GQs by the 50 RGG conformations (Fig. S3 in SI). The maps also clearly distinguish the mode of binding of RGG with RNA and DNA GQs. The RGG dominantly encounters the solvent-exposed external G-quartets of TERRA RNA in an “end stacking” mode. In contrast, it binds mostly in the grooves and loops of DNA GQs.

Interestingly, the binding modes influence the relative binding strength of the complexes. The calculated HADDOCK scores for the complexes in end stacking mode, such as those involving TERRA6, TERRA12, and TERRA24, are relatively lower in the range of −85 to −65. Whereas in groove binding mode, as in tel6, tel12, and tel22 complexes, the binding is slightly better (in the range of −100 to −75), with tel12 showing the strongest binding owing to the mixed mode of interactions. Another significant observation is that the HADDOCK scores for complexes involving tetra-molecular GQs (TERRA6, tel6) without propeller loops are weaker than those for GQs with propeller loops (such as TERRA 12, TERRA24, tel12, and tel22). These results corroborate well with experimental findings that the RGG primarily interacts with the GQ loops (11, 12). Our atomic resolution complex structures further provide deeper insights into the residue-wise contributions to the inter-molecular binding. In Tables S1 and S2, we provide a detailed list of inter-molecular interactions for the three strongest RGG-GQ complexes, screened by their HADDOCK scores. Intriguingly, in all these complexes, we observe over-whelming contributions of Arg and Gly from the RGG repeat motifs to Hydrogen bonding and electrostatic interactions, emphasizing the importance of RGG repeats in nucleic acid recognition. Also, the interspersing aromatic residues such as Tyr237, Tyr244, and several Phe residues are mediating π-stacking interactions. We also note a larger contribution of Ser residues in the DNA complexes.

### Both DNA and RNA GQ with loops and overhangs have stronger binding consistent with recent biochemical observations

**Figure 3:**
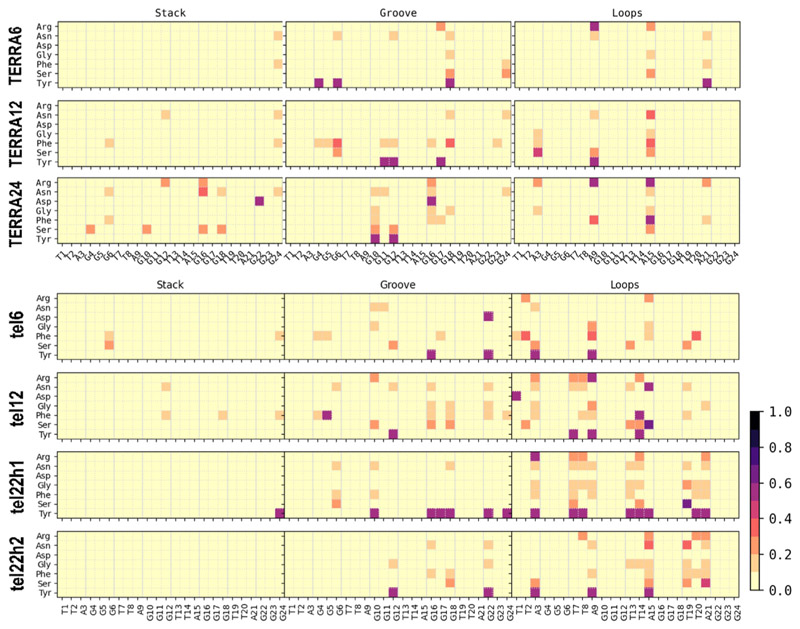
Binding modes of RGG with GQ. The normalized number of interactions between each amino acid type in RGG (y-axis) with every base of GQ (x-axis) was calculated from the simulated complexes. The interactions were calculated based on the distance between heavy atoms and the number of interactions was normalized on the total number of each amino acid type. Rows represent the seven GQ systems, while the columns represent end-stacking, groove, and loop-binding modes. The contact probabilities are color-coded on a *yellow* to *black* scale where darker colors indicate a higher probability of interactions.

**Figure 4:**
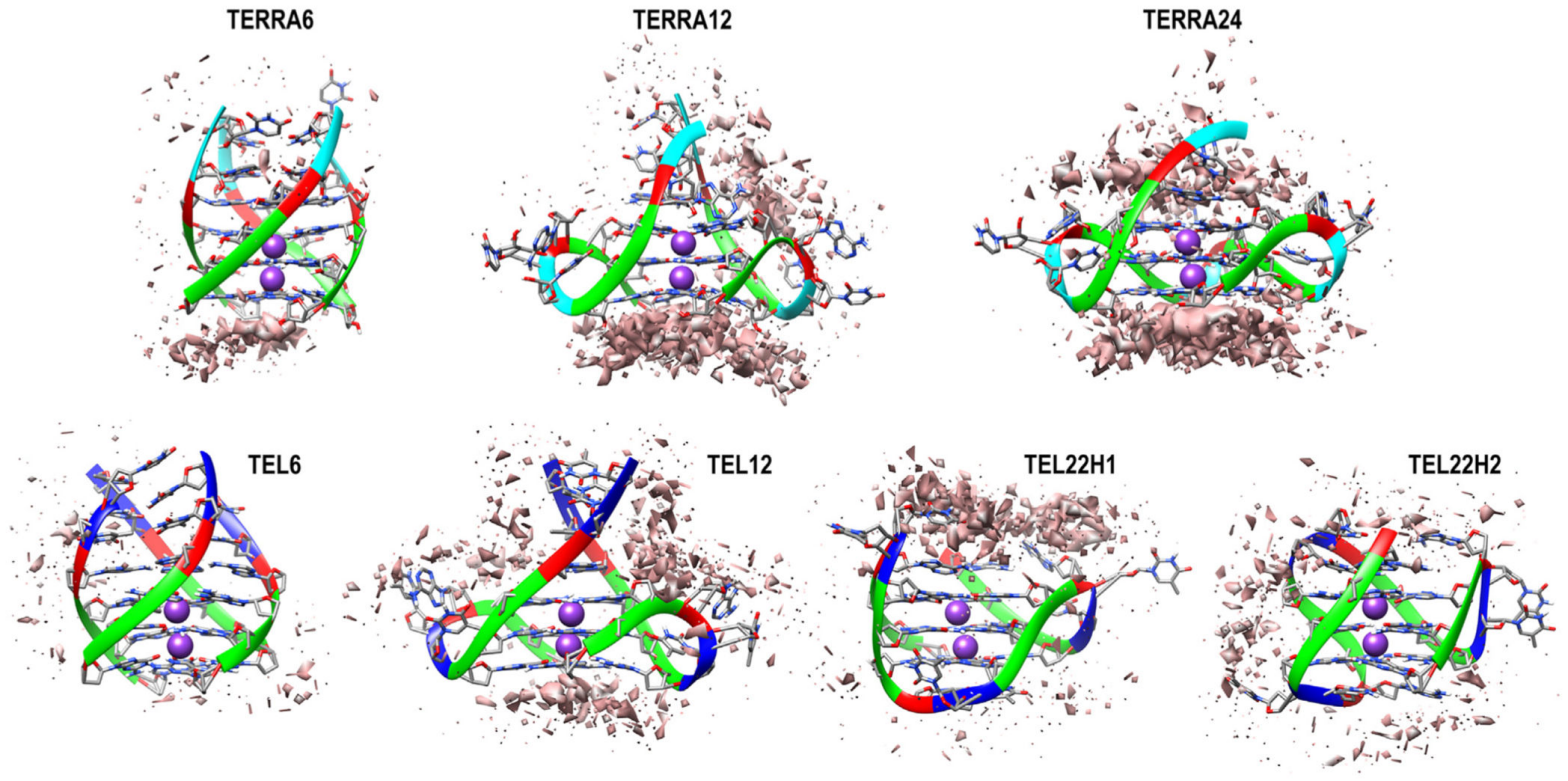
Occupancy map depicting the extent of RGG-GQ interaction after the 50 ns simulation. A high density of interactions at specific sites on GQ is shown as an isosurface mimicking the first solvation shell (*orange* surface). The interacting sites and density vary considerably from the complexes before simulation (see Fig. S3 in SI for occupancy maps before simulation). The GQ backbone is shown as ribbons while the bases are displayed as sticks. The bases are colored as *Green*: Guanine, *Red*: Adenine, *Blue*: Thymine, and *Cyan*: Uracil.

All the encounter complexes obtained from HADDOCK were simulated for a period of 50 ns each (a total of 17.5 μs of all-atom simulations) to monitor the variations in RGG binding orientation and its induced changes in the GQ structural stability. These simulation trajectories clearly revealed the dynamic changes in the binding modes of RGG with the GQs and the type of stabilizing interactions. Figure 3 depicts the inter-molecular interactions between the amino acids in RGG and each base of GQ. These interactions are normalized with the individual amino acid composition and indicate the participation of each amino acid type toward the stability of the complex. When an amino acid in RGG resides within 4 Å of the central Guanine O6 atom, it is considered to explore the end-stacking mode. Groove binding mode is identified when an amino acid of RGG forms contact with any of the atoms exposed in the grooves, like “N2”, “C2”, “N3”, “C4”, “N6”, “C8” or “N9” atoms of Guanine. Similarly, the loop binding mode of RGG is described by the interaction with TTA, irrespective of its backbone, sugar or base. The three binding modes are not exclusive, and a given conformation of RGG might exhibit all three binding modes simultaneously by different regions of RGG. The encounter complexes from docking studies clearly show the end stacking mode of TERRA12, TERRA24 and tel12, and groove or loop binding modes in other GQ complexes (Fig. S4 in SI shows occupancy maps before simulation). However, after simulations, the binding modes vary, and groove or loop binding modes are preferred in all GQ complexes (Fig. 4).

### Simulated ensemble of RGG-GQ complexes show interactions consistent with NMR experiments

**Figure 5:**
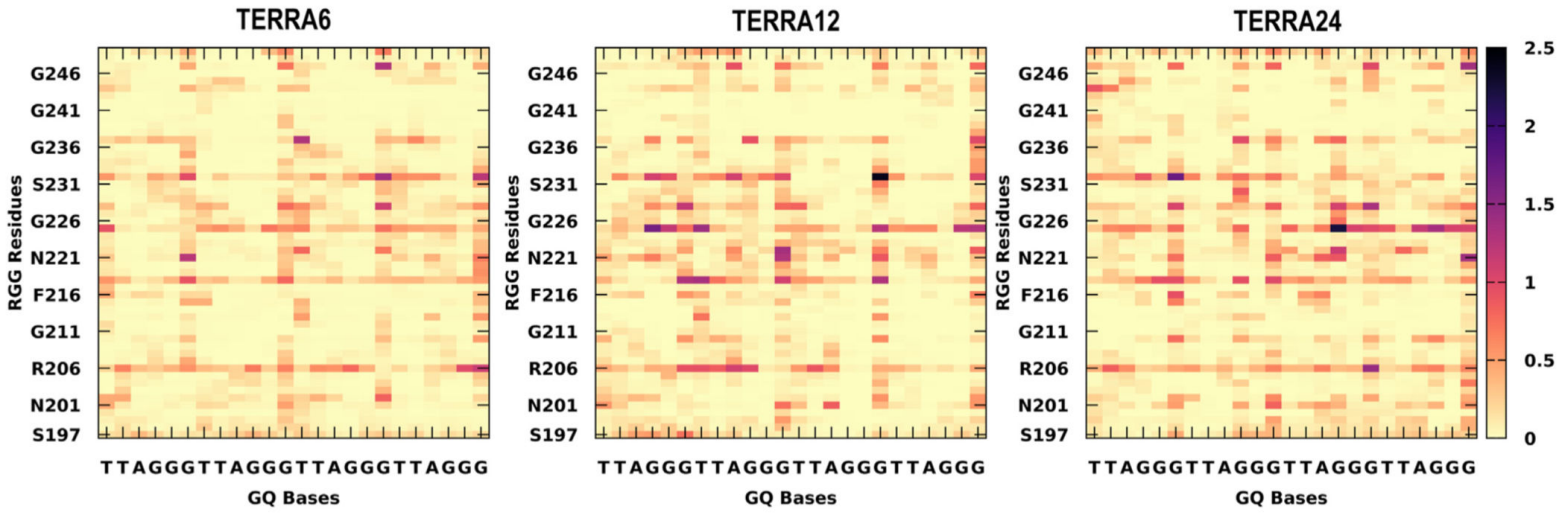
Residue-wise contact maps representing telomeric RNA-GQ interactions. The x-axis indicates the GQ bases and the RGG sequence on the y-axis. The color scale varies from *yellow* to *black* and indicates a longer lifetime of interactions. The contacts were calculated with a distance cutoff of 4 Å.

**Figure 6:**
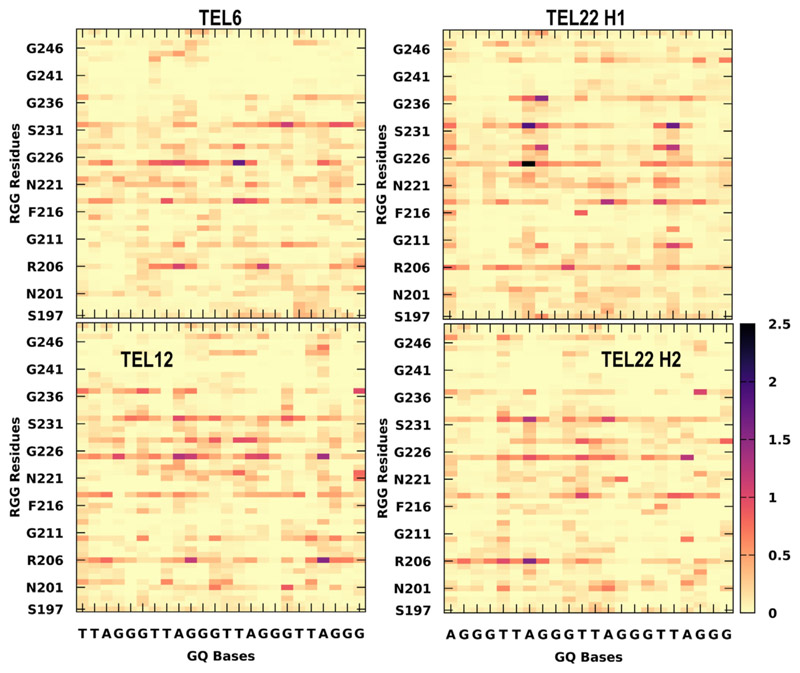
Residue-wise contact maps representing telomeric DNA-GQ interactions. The x-axis indicates the GQ bases and the RGG sequence on the y-axis. The color scale varies from *yellow* to *black* and indicates a longer lifetime of interactions. The contacts were calculated with a distance cutoff of 4 Å.

In the previous section, we delineated the RGG binding sites in GQ and the preference of each amino acid type to interact with specific binding sites. Following this, we explore the lifetime of all RGG-GQ interactions by generating contact matrices (Figs. 5 and 6). The lifetime of RGG-GQ interactions in TERRA complexes is higher than in DNA GQ complexes. Preliminary inspection of these contact maps clearly shows that the N- and C-termini of RGG do not exhibit strong interactions. Previous NMR studies have highlighted the role of Tyr244 in binding GQs. Other residues of RGG like Ser197, Ser231, Arg232, Gly233, Tyr244, Asn245, and Gly246 were also reported to show chemical shift perturbations upon binding with GQ (11, 12, 46). Our contact analysis shows that these residues interact strongly with GQs, along with several other residues, particularly in the core of RGG (between Phe216 to Arg232). Altogether, by using our protocol, we were able to generate protein-nucleic acid complex structures that match well with experimental data and also provide additional atomic-level insights into the structural features.

### RGGs can perturb both RNA and DNA GQs

**Figure 7:**
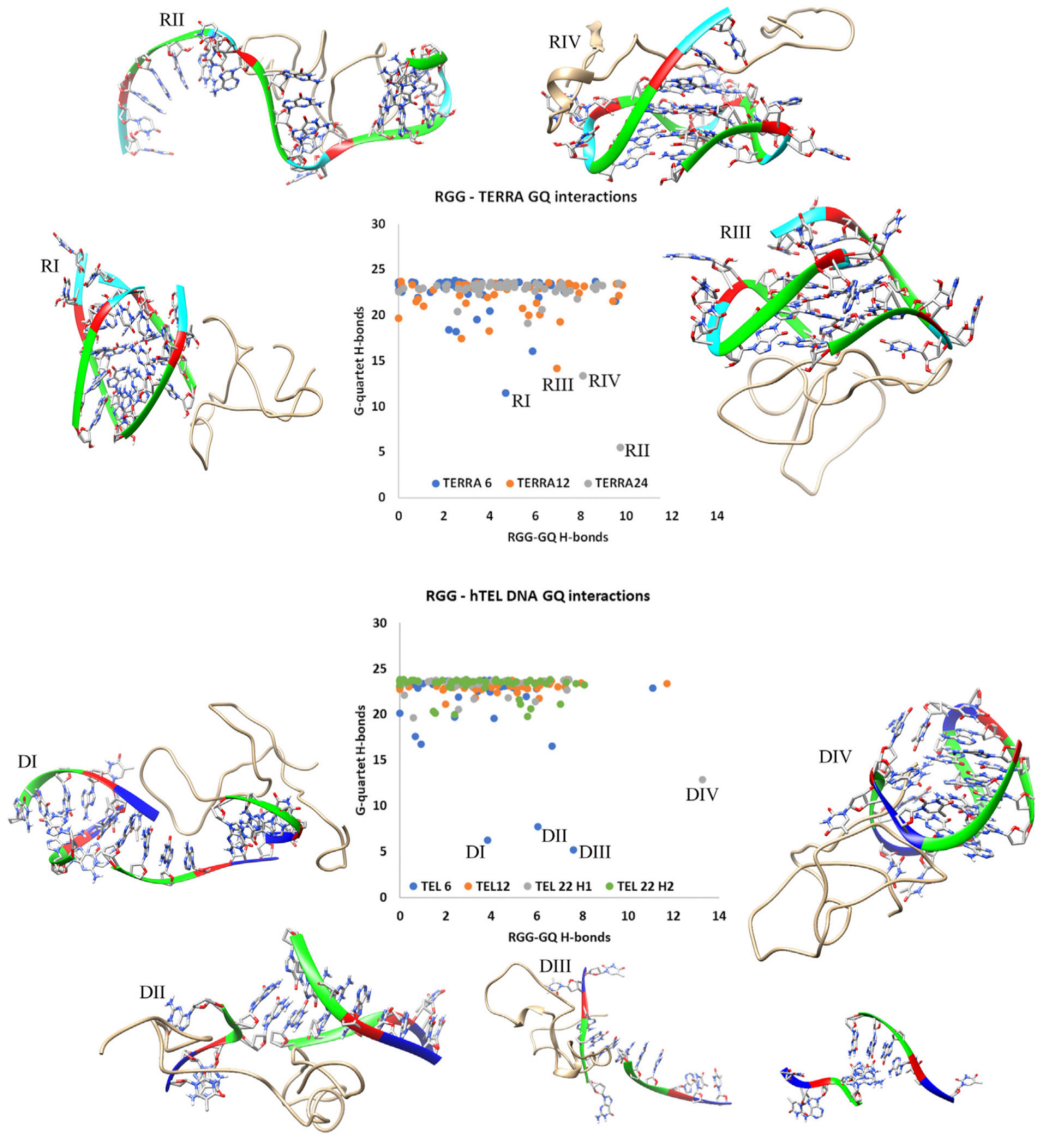
Scatter plot between the number of intermolecular RGG-GQ (RNA and DNA) hydrogen bonds and intramolecular G-quartet hydrogen bonds highlighting the complex stability. The unfolding complexes are labeled and the structures after 50 ns MD are shown.

Among the 350 simulated complexes, visual inspection shows that few of the GQ complexes were unstable. Hence, before analyzing these unstable complexes, we ensured the stability of the GQs by simulating their unbound conformations. The human telomeric repeat sequence has three consecutive guanines (d(TTAGGG)_n_), and consequently, each GQ topology chosen in our study has three G-quartets. Each G-quartet is stabilized by 2 Hoogsteen H-bonds between adjacent guanines and a total of 8 H-bonds per quartet. Collectively, the three quartet GQs are stabilized by a minimum of 24 H-bonds. Our apo-state simulations show that all seven GQ structures were stable for the entire simulation period of 300 ns as seen by the time evolution of their G-quartet hydrogen bonds (Fig. S5 in SI). Hence, any changes in the stability of GQs in the RGG bound complexes are considered as the effect of RGG binding. During simulation, the RGG remains dynamic and experiences changes in its conformation as well as its binding with GQ. Considering the stability of apo GQ structures and the dynamic behavior of RGG, if a 50 ns simulation time induces any local disruptions in the GQ structure, it can be interpreted as a direct effect of RGG binding. The average number of hydrogen bonds between RGG and GQ as well as within the G-quartets were calculated in all the simulated complexes and a scatter plot correlating these is shown in Figure 7. A majority of the GQs possess 22 ± 2 hydrogen bonds and hence, they are stable. Loss of even two hydrogen bonds in each of the three G-quartets would elicit destabilization in the overall GQ structure. Therefore, we used an arbitrary cut-off of 15 hydrogen bonds or less (of the total 24) within the G-quartets to consider it as unstable. Figure 7 shows that at least one among each of the three types of TERRA complexes is unstable. Similarly, in the case of the telomeric DNA (Fig. 7), three among the 50 tel6 and one among the 50 tel22h2 complexes are unstable. Snapshots of these complex structures after the 50 ns molecular dynamics (MD) simulation are also shown in Figure 7.

### The binding mode of RGG defines the mechanism of RGG-induced perturbation of GQs

**Figure 8:**
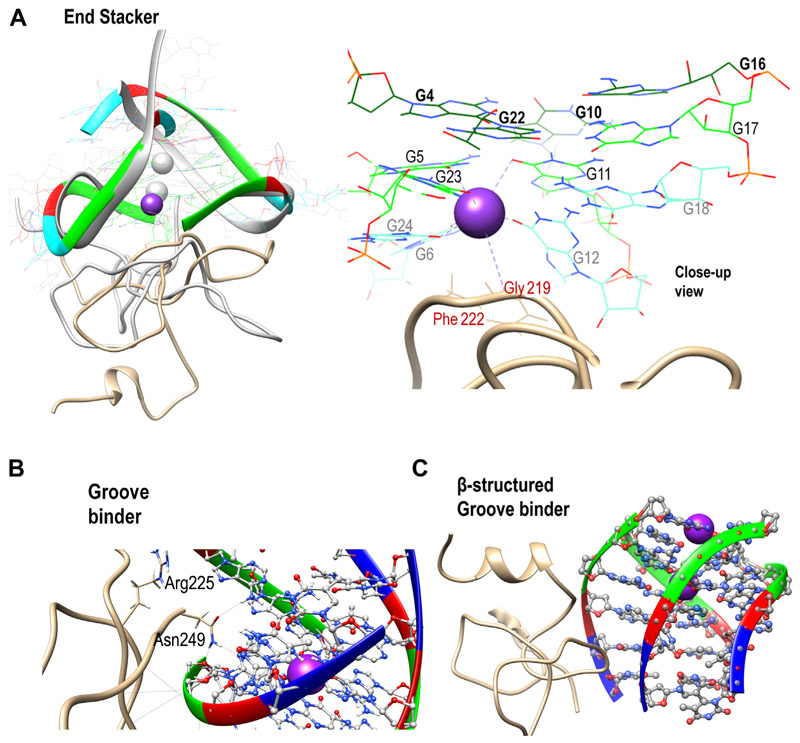
RGG-induced unfolding mechanism. *A*, the end stacking mode of TERRA12 and (*B*) the groove binding mode of tel6 are shown as examples. The superposition of the initial structure (in *grey*) and 50 ns simulated conformations of TERRA12 are shown to clearly portray the loss of coordinated K+ ions due to RGG. *C*, depicts the unfolding of tel6 by a β-structured motif binding in the groove. The RGG backbone is shown as ribbons while the sidechains are depicted in wire representation. The K+ ion is shown as a *purple* sphere and its coordination shell is identified with dotted lines. Phosphate: *Yellow*, Carbon: *gray* (RGG)/*brown* (GQ), Nitrogen: *Blue*, Oxygen: *red*, Hydrogen: *white* and K+ ion: *purple*.

About 2% (8 out of the 350 complexes) of the simulated RGG-GQ compelxes show perturbation in the GQ structure upon binding with RGG. Interesting mechanistic insights into RGG-induced GQ destabilization were observed by analyzing the trajectories of these unstable complexes (a detailed discussion is included in Annexure 1 in SI). Instabilities in GQ can be induced by two major mechanisms like perturbing the core-bound ions or the grooves. One of the TERRA12 complexes which is unstable with only 15 G-quartet H-bonds, binds RGG in the end stacking mode (labeled RIV in Fig. 7). By visualizing this trajectory, we clearly observed the expulsion of K_+_ ion by RGG (Fig. 8*A*). A π-stacking interaction by Phe222 stabilizes the G12, while the Gly219 is positioned to intrude into the core of GQ, leading to the repositioning and expulsion of a K_+_ ion in the core. We have shown in the previous sections that the RGG domain has a propensity to form beta-structures and turn-like structures. Our simulations show that these turn-like structures can stack over the G-quartets *via* π-stacking interaction between Phe and Guanine bases and cause perturbations in the core ions. To validate this RGG structure-induced GQ perturbation hypothesis, we trimmed the RGG to retain only the 205 to 219 region in one of the TERRA24 complexes and simulated this truncated RGG-GQ complex. During the simulation, the peptide continues to interact with the GQ *via* Phe-Guanine stacking and induces the displacement of K_+_ ions from the GQ core. Interestingly, the Phe residues responsible for π-stacking interaction are a part of the FGG motif of hnRNPA1.

The tetramolecular structures are particularly easy to unfold by interactions at the grooves. In this mode of binding, the side chains of RGG amino acids are oriented to interact with the backbone and nucleobases of GQs, straining the GQ to unfold. In one of the tel6 complexes (labeled DIII in Fig. 7), the sidechain of Asn249, interacts directly with an oxygen atom in the deoxyribose sugar of G5 and N3 atom in the Watson-crick face of G4 (Fig. 8*B*). These interactions are due to the protrusion of the Asn sidechain into the groove between two parallel strands of DNA, thereby disintegrating the tetramolecular GQ through the grooves. The ability of Asn to form bifurcated H-bonds is well-known and this is the major reason for Asn-induced GQ perturbation. We mutated this Asn into Ala (to prevent H-bond formation) or Arg (to mimic bifurcated H-bonds) and simulated the two complexes to reinforce the role of Asn in perturbing the GQ structure. In the case of Ala mutation, the tel6-GQ remained stable, whereas in the case of Arg mutation, the tel6-GQ was perturbed indicating that the interaction of these amino acids at the grooves of GQ might cause perturbations. It is important to note that these Asn residues are in the vicinity of the Phe residues forming the FGG motif. The stability of complexation due to the stacking of Phe over the G-quartets might allow the Asn to establish multivalent H-bonds in the GQ grooves, leading to GQ perturbation. Altogether, our observations indicate that the orientation of amino acid sidechains and their ability to form β-structured motifs induces destabilization in the GQ structure irrespective of their binding modes, strengthening the possibility of an RGG-induced mechanism for destabilizing GQs.

It has been shown previously that the GQ unfolding rate of the UP1 domain (k_obs_ = 0.63 ± 0.01) is lower than UP1+RGG (k_obs_ = 1.93 ± 0.01) (11). Our study supports this observation and provides a structural basis for the earlier biochemical observation. Since we show that the individual RGG domain is capable of causing perturbations in the GQ topology, it could be a possible mechanism of catalyzing the unfolding activity of UP1. The observation suggests that RGG not only acts as an adaptor for recognizing GQ but also could play a crucial role in expediting the GQ unfolding.

## Conclusion

IDRs and IDPs are shown to exhibit a variety of functions by exploiting their inherent disorderliness. They form unique molecular signatures to specifically cater to a definite function. These molecular or structural signatures can be the formation of a particular secondary structure upon binding with a folded partner (55) or retain the disordered state to recognize the folded partner (56–59), and in few cases, express non-specific interactions with another disordered molecule (53). Such diversity in their structures is necessary to fulfil their diverse functions by recognizing different biomolecules. One such IDR is the RGG-rich domain, which has the ability to bind proteins and nucleic acids. The nucleic acid binding property of RGG-rich regions is well known, and the RGGs of several functionally important IDPs like hnRNPA1, FMRP, and FUS express either stabilizing or destabilizing effects on GQ motifs (16, 60). It is intriguing that the RGG domains of different proteins show differential binding with G-quadruplexes, and some RGG like that of TDP-43, are able to selectively bind to the parallel form of telomeric G-quadruplex. In regard to the sequence properties, Glycine residues are proposed to interact with the nucleic acid based on the activity of the RGGGGR peptide of FMRP (61). Also, binding studies on FMRP RGG-box with an RNA duplex-quadruplex junction revealed a well-formed β-turn in RGG is responsible for its binding to the RNA (62). A similar involvement of β-spiral structure in FUS is reported as necessary for binding a GQ motif (63). Though the importance of sequence and secondary structures like β-motifs to bind nucleic acids, particularly GQ motifs, is known, a high-resolution mechanistic detail is still lacking. Our study is an attempt to faithfully capture a high-resolution conformational landscape explored by the RGG domain of hnRNPA1 through advanced simulations and further explore its functional significance.

Our hybrid replica exchange method has been able to successfully generate the conformational states attained by RGG in a solution that also matches well with the chemical shifts of an NMR ensemble. Beyond matching the chemical shifts, our generated conformation ensemble showed secondary structures similar to their propensities in the experimental ensemble. The conformation ensemble generated in our study shows a high propensity for β-turns and transient but recurring β-sheets. Our docking study highlights that these turn-like structures can stack on the G-quartets and they can be accommodated well in the grooves of GQ. Although the RGG domain of hnRNP A1 is called the “RGG-rich” region, this region is richer in FG/FGG motifs. Our study shows an important contribution for these Phe and Asn to facilitate the localization of RGG to GQ (stacking of Phe over the G-quartets) and induce perturbation (by bifurcated H-bonds at the GQ grooves) in the GQ structure. The individual RGGs were able to perturb the GQs, although less frequently (∼8 events out of the total 350 complexes generated). This perturbation of GQ by the RGG of hnRNP A1 should be attributed to a combination of events like the stacking of Phe due to their exposure in a turn-like structure, and the consequent orientation of Asn sidechain at the GQ grooves.

The RGG of hnRNPA1 is reported to enhance the unfolding of intramolecular human telomeric GQ by UP1 during telomere maintenance. The primary recognition site of RGG is reported as the bases forming the propeller loops of unimolecular GQs. The enhanced GQ unfolding by UP1 in the presence of RGG can be attributed to two different possibilities. In the first case, the RGG might act as the initial recognition factor, binding stably with the GQs and recruits UP1. Along with UP1 recruitment, the RGG can also induce slight perturbations in the GQ structure, thereby facilitating an easier and efficient binding of UP1. Based on our extensive docking and simulations, we propose the possibility of RGG-induced GQ perturbation. The concentration of hnRNPA1 and GQs, along with physiological conditions, play a vital role in molecular recognition since the RGG conformations and GQ topologies are highly dependent on these factors. Altogether, in this study, we have presented a protocol for generating a high-resolution conformation ensemble of an IDP (the RGG domain of hnRNPA1), capturing the heterogeneity and exploring their functional significance.

## Methods

### Conformer generation of RGG

The initial unfolded structure of the RGG domain was obtained from Iterative Threading ASSEmbly Refinement (I-TASSER) which is a widely used server for automated protein structure prediction and structure-based functional annotation (64–66). Using the amino acid sequence as a starting point, I-TASSER constructs 3D structural models. The generated structure of the RGG domain is a collapsed coiled-coil structure. The usage of I-TASSER is limited to generating initial coordinates of the protein and further conformation sampling was carried out using molecular dynamics (MD) simulations.

For IDPs, computational methods can be used to generate a conformation ensemble to study their functions. Some of the commonly used, less computationally demanding methods are flexible-meccano (67), BUNCH (68), and IDP-ConformerGenerator (69), among others. These methods use backbone dihedrals of non-secondary structural elements compiled from the existing protein structures, folded or disordered. Based on prior knowledge, propensities for secondary structures or long-range interactions can also be included as criteria in these models. However, the most commonly used conformer generation methods are based on molecular dynamics or Monte Carlo simulations. In general, coarse-grained models (70, 71) are used to reduce the time complexity over all-atom models, however, these force fields have several limitations to sample IDPs. We have developed an advanced sampling technique called replica exchange with hybrid tempering (REHT) to generate faithful conformations of IDPs (43). This method has been used to generate all-atom models and has been tested extensively to generate conformation ensembles of several IDPs like Histatin five and α-synuclein. We have used this REHT method to generate the conformation ensemble of RGG in this study.

### REHT simulation

Unlike the commonly used temperature-based replica exchange, our hybrid approach involves the exchange of both the temperature as well as the Hamiltonian across the replicas. The protein was solvated using the 3-site rigid TIP3P water model in a cubic box with a minimum distance of 1 nm from the surface of the protein. The systems were also neutralized to maintain a physiological concentration of NaCl (0.15 M). The Charmm36 m (72) forcefield was used to model the protein. The simulations were carried out using Gromacs-2016.5 patched with Plumed-2.4.1 (73–75). Initially, the modeled protein was energy minimized using the steepest descent algorithm for 50,000 steps to avoid any poor contacts. The energy-minimized structure was then thermalized and equilibrated sequentially in NVT and NPT ensembles for 1 ns.

Next, the protein and the solvent were coupled separately to the target temperatures using the Nose-Hoover thermostat, and the final production simulation was performed in the NVT ensemble. A cut-off of 1 nm was used for calculating the electrostatic and vdw interactions and Particle Mesh Ewald was used for long-range electrostatics. To integrate the equations of motions, the Leap-frog integrator with the time step of 2 fs was used. LINCS algorithm was used to constrain all the hydrogen atoms. Exchanges were attempted at every 1 ps interval. In this approach, the Hamiltonian of the solute particle in the different replicas is scaled down to 0.6 to 1 while also raising the temperature of the systems simultaneously up to a maximum of 500 K. This way, as a function of both lambda scaling and explicit thermostat conditions, a very high temperature is ensured to be realized on the protein solute to sufficiently overcome the energy barriers. The solvent is heated up mildly in order for the rapid reorientation of the hydration shell upon conformational change of the protein. 20 replicas were used to simulate the RGG in a temperature range of 300 K to 500 K for a cumulative period of 1 μs and an acceptance probability of 0.3. Post-processing analyses of the trajectories were performed with the Gromacs analysis tools and the trajectories were visualized using VMD.

### Chemical shift calculation

We have used SPARTA+(version 2.90) in conjunction with a software package called Mdtraj (76, 77). SPARTA + employs a well-trained neural network algorithm to construct quantitative relations between chemical shifts and protein structures, including backbone and side-chain conformation, H-bonding, electric fields, and ring-current effects. Its single-level feed-forward multilayer artificial neural network (ANN) is capable of identifying the dependence of ^15^N^H, 13^CO, ^13^C_α_, ^13^C_β_, ^1^H_α_, ^1^H^N^ chemical shifts on the local structural and dynamic information as well as amino acid type, and those of its immediate neighbors. Mdtraj is a Python software package that is designed to analyze trajectory data generated from MD simulations.

We have compared our predicted data with the experimentally calculated chemical shift submitted in the Biological Magnetic Resonance Data Bank (BMRB entry 50017) (46, 78). For comparison purposes, we have used secondary chemical shift data by removing random coil chemical shifts from the raw chemical shift. For ΔCα-ΔCβ calculation, the secondary chemical shifts of ^13^C_α_ and ^13^C_β_ are subtracted to do away with the referencing error, if there is any.

### Secondary structure propensity calculation

We have used the secondary structure prediction algorithm (SSP) developed by Prof. Julie D. Forman-Kay’s group (48). We have used ^13^C_α_, ^13^C_β_, and ^1^H_α_ chemical shifts for this purpose as recommended by the algorithm developers in the context of intrinsically disordered proteins. For random coil and secondary structure chemical shifts and standard deviations, we have used RefDB. All the data have been re-referenced.

### t-SNE clustering

We have chosen a nonlinear dimensionality reduction method called t-distributed Stochastic Neighbor Embedding (t-SNE) in order to separate high-dimensional heterogeneous IDP ensembles into meaningful clusters. An important aspect of the t-SNE algorithm is the perplexity value, a tuneable parameter in t-SNE that balances the information between the local and global features of the dataset under investigation. Diligent choice of perplexity value for a given data is important to be able to most discretely divide the data into unambiguous clusters. In our work, we have noticed that for very low perplexity values, the clustering is extremely diffusive in nature and for a high value of perplexity parameter, the entire dataset seems to be treated as a single cluster. The performance of t-SNE is fairly robust to changes in the perplexity. In this work, we have explored several perplexity value options ranging from 50 to 2000. We have chosen the values that most discretely divide the data points into separate clusters. To aid the result of t-SNE clustering in further partitioning, we have employed a widely used centroid-based technique called K-means clustering which is one of the favored and standard unsupervised machine learning algorithms. We have used the SciKit tool for these analyses which is an open source library for machine learning in Python (79).

### Structures of RGG and GQ

The human telomeric DNA (htel) and RNA (TERRA) GQs, formed by the telomeric repeat d(GGG[T/U][T/U]A)n, is made by the stacking of three G-quartets connected by TTA or UUA loops. The Guanine bases in a quartet are stabilized by hydrogen bonds between their Watson-Crick and Hoogsteen edges forming a square planar arrangement. The central-facing carbonyl oxygen atoms of guanines exert electrostatic repulsion, which is shielded by the coordination of cations at the core of GQs. Polymorphism is a common feature in DNA GQs, where different GQ topologies are observed for a given sequence under different physiological conditions like the available cation types, neighboring G-rich sequence, and their interacting partners (80, 81). The human telomeric DNA GQs exhibit structural polymorphism with a wide range of topologies varying from tetramolecular or bimolecular all-parallel quadruplex to unimolecular all-parallel, anti-parallel as well as hybrid forms. Also, the variation is evident with the presence of different cations like Na_+_ and K_+_. However, due to the presence of ribose sugar, TERRA GQs can adopt only a parallel GQ coordinated by K_+_ ions (2).

The 50 conformations of RGG obtained by clustering the REHT trajectories were docked with the human telomeric DNA and RNA GQ (also known as GQ). Unimolecular, bimolecular, and tetramolecular forms of both DNA and RNA quadruplexes were modeled in this study (Table 1). The tetramolecular TERRA (or TERRA6) was modeled using the solution structure with PDB ID: 6GE1 (82), while the unimolecular TERRA24 was modeled based on the telomeric DNA quadruplex with PDB ID: 1KF1 (83). The NMR structure of bimolecular telomeric RNA (or TERRA12) is available in the Protein Databank with the ID 2KBP (84). The structures of all forms of telomeric DNA quadruplexes were taken directly from the Protein Databank with IDs 1NP9 (tetramolecular or tel6) (85), 1K8P (bimolecular tel12) (83), 2HY9 (unimolecular form one tel22h1) (86) and 2JPZ (unimolecular form two tel22h2) (87). The unimolecular telomeric DNA in K_+_ ion solution adopts two different hybrid conformations that are equally populated and both the structures (differentiated as tel22h1 and tel22h2) were taken for our docking studies. The numbers behind TERRA* or tel* indicate the length of each RNA or DNA strand forming the GQ. Additional bases were added to the 5’ or 3’ ends of the GQ structures to match the sequences used in ITC studies by Ghosh *et al*. (11, 12). Two K_+_ ions were placed manually between the three G-quartets of all 7 GQs. The modeled structures were simulated for a period of 300 ns and the structures extracted after 10 ns simulation were used as starting structures for docking studies.

### HADDOCK docking protocol

HADDOCK (88) utilizes a data-driven approach for molecular docking. The stand-alone version of HADDOCK 2.4 was used to drive the docking of RGG with telomeric RNA and DNA GQs. Since the RGG conformations are randomly coiled structures of ∼53 amino acids, all residues are highly solvent-exposed. To perform an unbiased docking study, the distance between the center of mass of the protein and GQ alone was used as a restraint criterion. The docking process is performed in three steps: rigid-body docking, semi-flexible refinement, and water refinement. About 10,000 complexes were generated during the first stage of rigid-body docking by using the standard HADDOCK protocol. Among these, 400 lowest-energy structures were selected for subsequent semi-flexible simulated annealing and explicit solvent (water) refinement, to optimize the interface. HADDOCK scoring was performed according to the weighted sum (HADDOCK score) of different energy terms, including the van der Waals energy, electrostatic energy, distance restraints energy, inter-vector projection angle restraints energy, diffusion anisotropy energy, dihedral angle restraints energy, symmetry restraints energy, binding energy, desolvation energy, and buried surface area. The final structures were clustered using the fraction of common contacts (FCC) with a cutoff of 0.6 and a minimum cluster size of 4.

### Simulation of RGG-GQ complexes

For interaction studies of RGG-GQ complexes (listed in Table 1), we performed molecular dynamics simulation using Gromacs-2020.4 (89). We used a99SBdisp forcefield of Paul Robestelli for RGG, OL15 force field for DNA quadruplexes, and OL3 force field for RNA quadruplexes (90–92). The RGG-GQ complex systems (obtained from HADDOCK) were solvated with TIP3P water in a periodic box extending up to 1 nm and 1.2 nm in all directions respectively. The systems were neutralized and additional ions were added to mimic a salt concentration of 0.15 M. The short-range interactions were truncated with a cut-off distance of 1 nm. Electrostatic interactions were treated by particle-mesh Ewald with a real space cut-off value of 1 nm. Bonds containing hydrogens were constrained using the LINCS algorithm. The solvated and neutralized systems were energy minimized using the Steepest Descent algorithm followed by an equilibration of 5 ns and subsequently production runs. The temperature and pressure of the systems were maintained at 310 K and 1 atm using the nose-hoover thermostat and Parrinello-Rahman barostat in an NPT ensemble. Post-processing analyses were performed with the Gromacs analysis tools, and CPPTRAJ module of AmberTools20 and the trajectories were visualized using Visual Molecular Dynamics (93), UCSF Chimera v1.13. RGG-GQ interaction matrices were consolidated by calculating all heavy-atom contacts within 0.4 nm that are present for at least 1% of the total simulation period for each GQ (50 complexes * 50 ns = 2.5 μs).

## Supplementary Material

Supplementary Material

## Figures and Tables

**Table 1:** Telomeric RNA and DNA structures modeled in this study

Systems	PDB ID	Simulated systems		Comments
		Apo (ns)	With the 50 conformers of RGG (ns)	
TERRA6	based on 6GE1	300	50	tetramolecular RNA
TERRA12	2KBP	300	50	bimolecular RNA
TERRA24	based on 1KF1	300	50	unimolecular RNA
Te16	1NB9	300	50	tetramolecular DNA
tel12	1K8P	300	50	bimolecular DNA
tel22h1	2HY9	300	50	unimolecular DNA hybrid 1
tel22h2	2JPZ	300	50	unimolecular DNA hybrid 2

For every GQ, there are fifty complexes.

## Data Availability

Input files needed to initiate molecular simulations and full trajectory data of all simulations considered in this work are available on our server for download. Server data can be publicly accessed *via* the Figshare repository: https://figshare.com/s/4eb0701bdde1af2c2711c.

## Abbreviations

FCC: fraction of common contacts
GQ: G-quadruplexes
IDPs: intrinsically disordered proteins
LCD: low-complexity domain
POT1: protection of telomeres 1
PrLD: prion-like domain
REHT: replica exchange with hybrid tempering
RGG: Arg-Gly-Gly tripeptide repeats
TLS: translocated in liposarcoma
UP1: unwinding protein 1.
