# Supplementary material for: The ribonucleoprotein hnRNPA1 mediates binding to RNA and DNA telomeric G-quadruplexes through an RGG-rich region
Source: J Biol Chem. 2025 Apr 8;301(5):108491. doi: 10.1016/j.jbc.2025.108491 (PMC7617716; doi:10.1016/j.jbc.2025.108491)
Supplement: Supporting information [file mmc1.docx]

**The ribonucleoprotein hnRNPA1 mediates binding to RNA and DNA telomeric G-quadruplexes through an RGG-rich region**

Sangeetha Balasubramanian^a+^, Irawati Roy^a+^, Rajeswari Appadurai^a,b^ and Anand Srivastava^a,*^

Equal contributions: Sangeetha Balasubramanian and Irawati Roy

^a)^ Molecular Biophysics Unit, Indian Institute of Science, Bangalore, Karnataka 560012, India

^b)^ Department of Biology, Indian Institute of Science Education and Research, Tirupati, Andhra Pradesh 517619, India

Email: [anand@iisc.ac.in](mailto:anand@iisc.ac.in)^*^


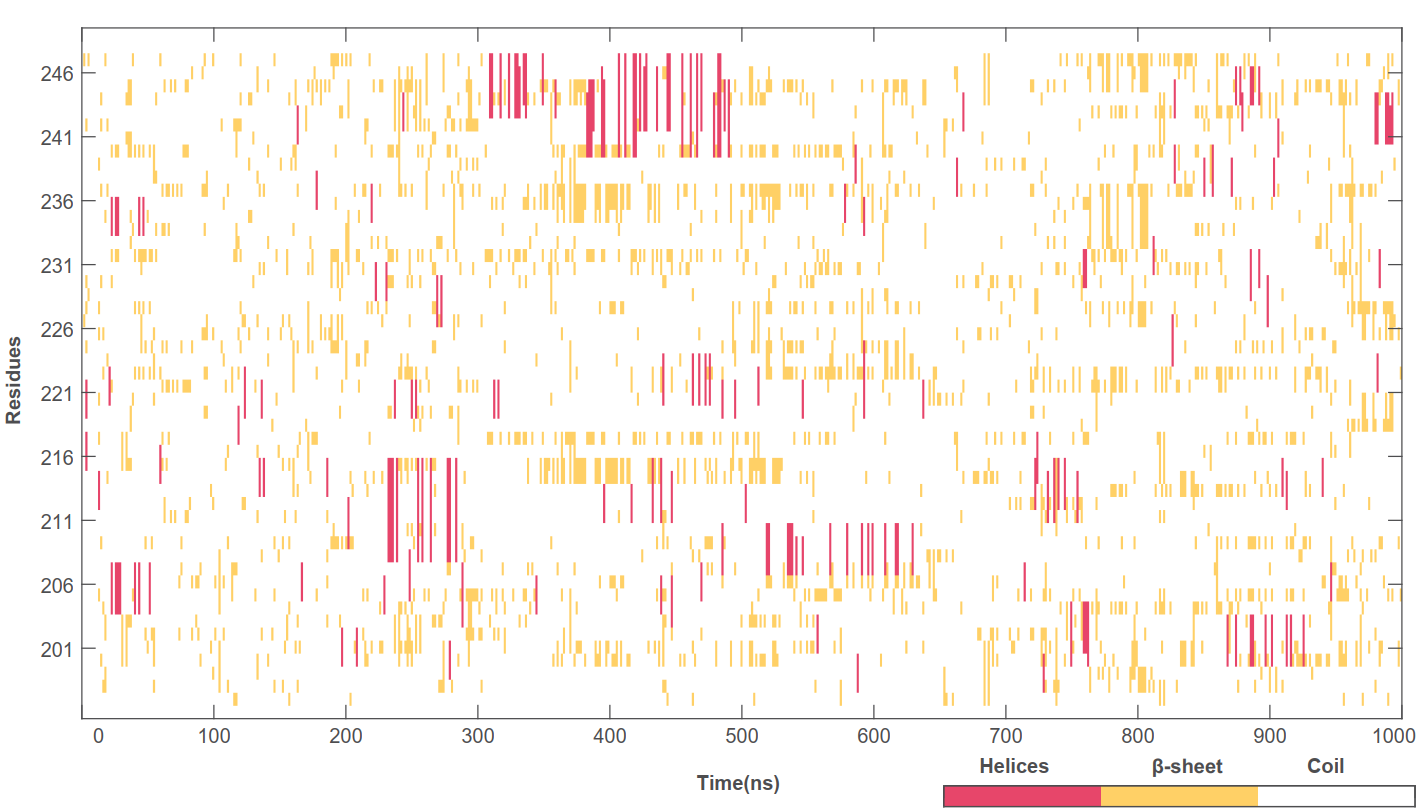


**Fig. S1:** Time evolution of secondary structures formed during simulation calculated using the DSSP algorithm. The time (1000ns) is plotted on X-axis. Y axis contains the residues. Relevant colour codes for the secondary structures are: White- Coil, Yellow- β-sheet, Red- Helices.


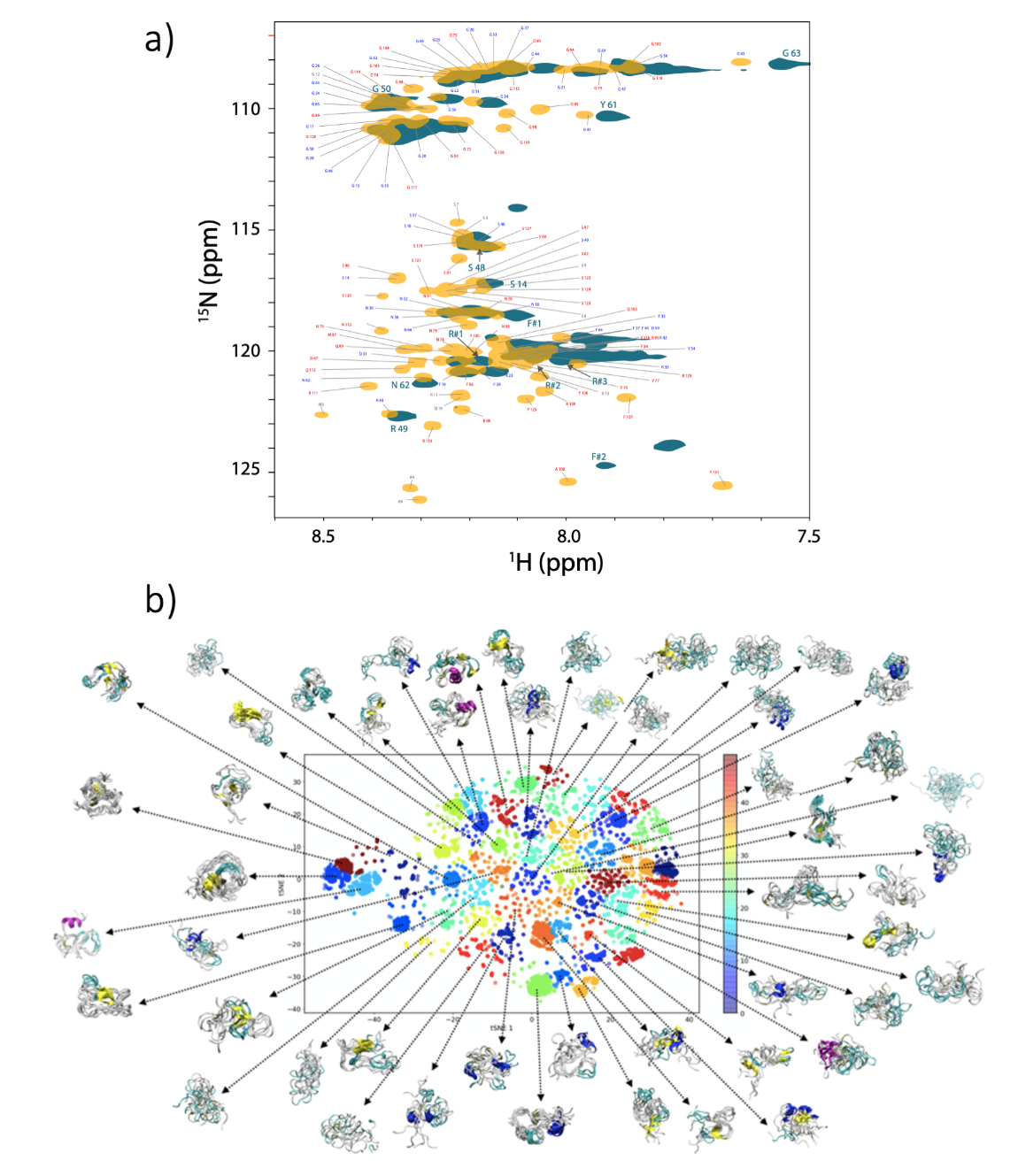


**Fig. S2:** a) Merged ^15^N^1^H HQSC plot. Yellow markers represents full hnRNPA1-LCD (46). Blue colored labels indicate RGG-box domain, red colored labels indicate PrLD domain. Teal markers represent only RGG-box domain represents (11). b) t-SNE clustering of RGG-box domain. The 50 clusters are color coded and representative structures from each clusters and superposed and mapped to the corresponding clusters.


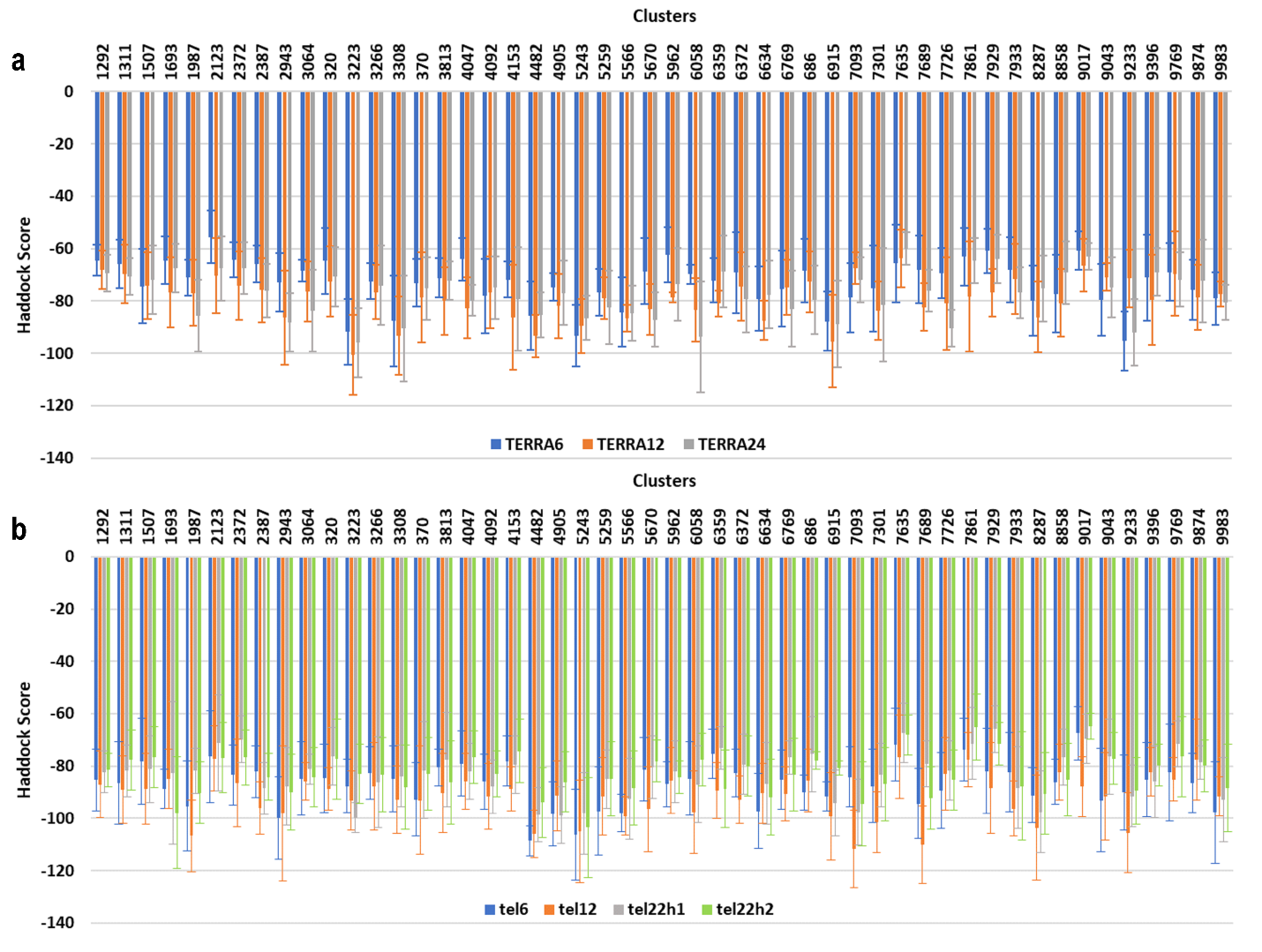


**Fig. S3:** The Haddock scores for each of the 50 RGG conformers used in the docking study with the seven telomeric G-quadruplexes (GQ), (a) tetramolecular (TERRA6 in Blue), bimolecular (TERRA12 in Orange) and unimolecular (TERRA24 in Gray) RNA quadruplexes. (b) tetramolecular (tel6 in Blue), bimolecular (tel12 in Orange), unimolecular form 1 (tel22h1 in Gray) and unimolecular form 2 (tel22h2 in Green) DNA quadruplexes.


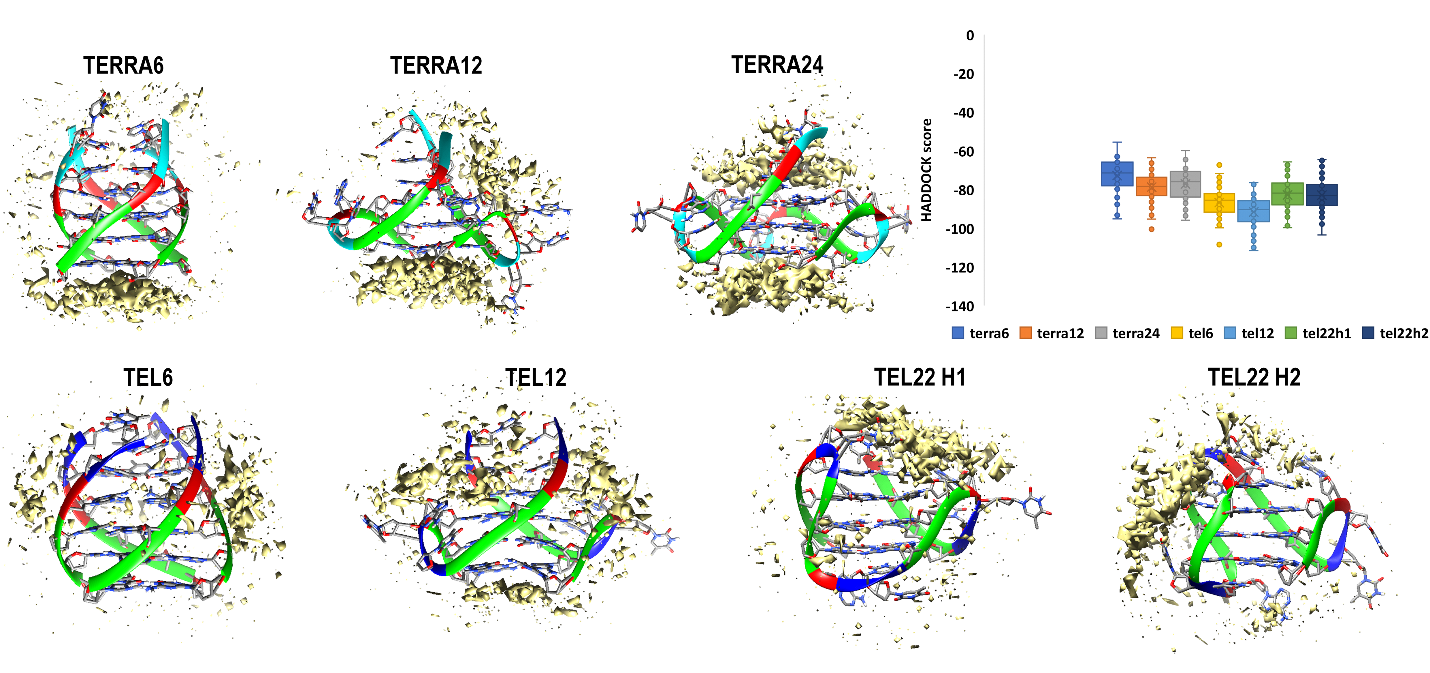


**Fig. S4:** Occupancy map depicting the extent of RGG-GQ interaction and the high density of interactions at specific sites on GQ are shown as an isosurface mimicking the first solvation shell (Yellow surface). The range of HADDOCK scores obtained for the docking of RGG with the seven GQ structures. Boxplots indicate minimum, median, maximum, and upper and lower quartiles. The GQ backbone is shown as ribbons while the bases are displayed as sticks. The bases are colored as Green: Guanine, Red: Adenine, Blue: Thymine and Cyan: Uracil.


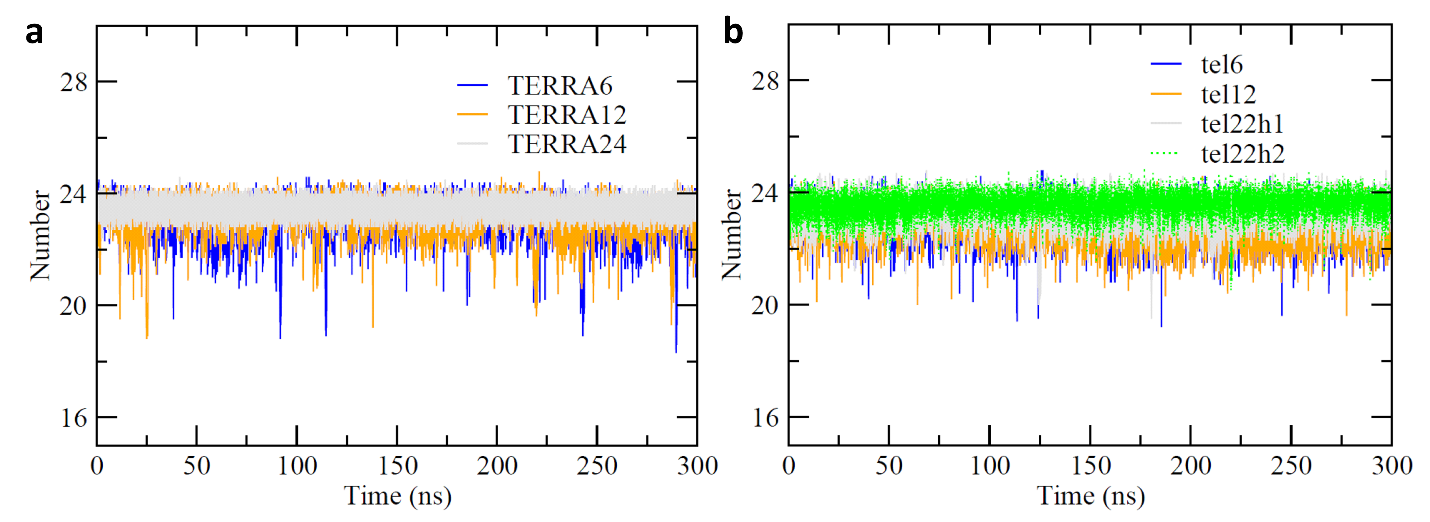


**Fig. S5:** Stability of free RNA (a) and DNA (b) GQ monitored by calculating the number of hydrogen bonds within the three G-quartets.

**Table S1.** RGG-RNA GQ interactions identified in the best three complexes screened by the HADDOCK score.

|  | **TERRA6** | | | **TERRA12** | | | **TERRA24** | | |
| --- | --- | --- | --- | --- | --- | --- | --- | --- | --- |
|  | **RGG conf.9233** | **RGG conf.5243** | **RGG conf.3223** | **RGG conf.3223** | **RGG conf.4482** | **RGG conf.6915** | **RGG conf.3223** | **RGG conf.6058** | **RGG conf.9233** |
| **Hydrogen Bonds** | SER197 - U2 | ARG225 - A15 | ARG225 - G18 | ARG218 - U19 | GLY205 - G4 | ARG225 - G5 | GLY211 - U7 | GLY198 - U19 | ASN215 - U14 |
|  | ASN215 - G5 | GLY226 - G23 | ASN249 - G11 | GLY220 - G24 | GLY208 - G23 | ARG225 - G4 | ASN215 - U7 | ARG218 - G12 | G6 - SER197 |
|  | PHE247 - U2 | ARG232 - G24 | ASN249 - G18 | SER223 - U7 | ARG225 - A3 | ARG232 - G18 | ARG218 - G6 | ARG218 - G12 |  |
|  |  | GLY233 - G24 | G18 - GLY248 | ARG225 - G12 | ARG225 - U2 | SER240 - G6 | ARG225 - G18 | ARG225 - G5 |  |
|  |  | G6 - GLY230 | G18 - ASN249 | GLY248 - G12 | SER231 - G4 | G24 - GLY226 | ASN249 - G23 | ARG225 - G6 |  |
|  |  |  |  | ASN249 - G17 | G23 - GLY208 |  | G24 - PHE247 | GLY229 - U8 |  |
|  |  |  |  | ASN249 - G12 |  |  |  | ASN249 - U13 |  |
|  |  |  |  |  |  |  |  | G6 - GLY227 |  |
|  |  |  |  |  |  |  |  | G24 - ASP 214 |  |
| **π-interactions** | A3-PHE202 | PHE247-G6 | PHE222-G6 | PHE222-G6 | PHE228-G10 | PHE216-U7 |  | ASP214-G24 | PHE202-G12 |
|  | A3-TYR244 | G22-PHE228 | G12-PHE247 | PHE247-G12 | PHE210-G5 | PHE228-G24 |  | U8-PHE228 | PHE202-G18 |
|  |  | G24-TYR244 |  |  |  | PHE247-G23 |  |  |  |
| **Electrostatic** | SER197,  ARG218,  ARG232 | ARG225, ARG232 | ARG225 | ARG218,  ARG225,  ARG232 | ARG225,  ARG232 | ARG218,  ARG225,  ARG232 | ARG218,  ARG225,  ARG232 | SER197,  ARG218,  ARG225 | SER197,  ARG206,  ARG218, ARG232 |

**Table S2.** RGG-DNA GQ interactions identified in the best three complexes screened by the HADDOCK score.

|  | **TEL6** | | | **TEL12** | | | **TEL22H1** | | | **TEL22H2** | | |
| --- | --- | --- | --- | --- | --- | --- | --- | --- | --- | --- | --- | --- |
|  | **RGG conf.4482** | **RGG conf.5243** | **RGG conf.9983** | **RGG conf.7093** | **RGG conf.7689** | **RGG conf.1987** | **RGG conf.3223** | **RGG conf.4905** | **RGG conf.4482** | **RGG conf.5243** | **RGG conf.1693** | **RGG conf.7093** |
| **Hydrogen Bonds** | GLY205 - T2 | ARG206 - G12 | SER197 - A9 | SER199 - T20 | GLY211 - G22 | ASN215 - G16 | ARG218 - T5 | GLY207 - T5 | GLY204 - G14 | GLY198 - G3 | ASN201 - A7 | ASN213 - T11 |
|  | ARG206 - T7 | ARG225 - T19 | SER197 - T7 | GLY209 - G24 | GLY220 - A15 | ARG218 - T14 | ARG218 - T6 | PHE210 - T11 | ARG206 - G15 | ASN215 - T5 | ARG206 - T6 | GLY236 - T6 |
|  | GLY224 - T2 | GLY226 - A3 | ASN221 - T2 | ASN221 - G22 | ASN221 - T14 | ARG218 - A15 | GLY219 - G8 | ARG225 - A7 | GLY208 - G21 | ARG218 - G4 | ARG206 - A7 | GLY239 - T5 |
|  | GLY227 - T19 | GLY227 - A3 | PHE228 - G10 | ARG225 - A3 | SER231 - G4 | ASN221 - G11 | GLY220 - G9 | GLY226 - A7 | GLY220 - G2 | PHE222 - T11 | PHE210 - T5 | SER240 - T5 |
|  | SER231 - T2 | SER231 - A3 | ASN245 - A9 | SER231 - G4 | ARG232 - A3 | ARG232 - G10 | GLY248 - T11 | SER231 - G8 | G14 - GLY204 | SER223 - T11 | ARG225 - T12 |  |
|  | SER231 - A3 |  | ASN249 - G17 | GLY235 - G4 | SER240 - A21 | GLY238 - G11 |  | ARG232 - G9 |  | SER231 - T6 | PHE247 - T6 |  |
|  |  |  |  | TYR 237 - A21 | SER240 - G22 | SER240 - G12 |  |  |  | GLY235 - G9 | G10 - GLY229 |  |
|  |  |  |  | GLY238 - G22 | GLY248 - G17 | SER240 - G11 |  |  |  | PHE247 - A13 |  |  |
|  |  |  |  | GLY238 - G23 |  | G4 - TYR 237 |  |  |  |  |  |  |
|  |  |  |  | T13 - GLY224 |  |  |  |  |  |  |  |  |
| **π-interactions** |  | G12-PHE247 |  | G16-PHE247 |  | G22-PHE222 | PHE202-T11 | PHE222-A7 | PHE228-A7 | PHE247-T5 | SER197-G2 |  |
|  |  | G5-PHE202 |  | T2-PHE228 |  | G22-TYR237 |  |  | G9-PHE202 |  | G10-TYR244 |  |
|  |  |  |  |  |  |  |  |  | G9- PHE210 |  | T12-PHE202 |  |
| **Electrostatic** | ARG206,  ARG225,  ARG232 | ARG225,  ARG232 | SER197,  ARG225 | ARG232 | ARG206,  ARG218,  ARG232 | ARG225 | ARG218, ARG225 | ARG206 | ARG225, ARG232 | ARG206, ARG225 | SER197, ARG218 |  |

**Annexure 1. GQ unfolding simulations**

Since a few unfolding events were witnessed in our study, we made an attempt to replicate these unfolding events and to test our hypothesis of RGG-induced GQ perturbation. Several trials were made, however, only a few simulations were successful in perturbing the GQs. This further reinforces that the RGG-induced perturbation is stochastic and hence only a small fraction of the simulated ensemble of RGG-GQ complexes were able to show instabilities. These simulations are listed below.

**Table A1.** Successful attempts (showed instability of G-quadruplex)

| **Complex** | **Time (ns)** |
| --- | --- |
| TERRA24-RGG truncated (205-219 AA) | 150 |
| tel22h2-RGG (Asn bound) | 20 |
| tel6-RGG (N249A) | 20 |
| tel6-RGG (N249R) | 20 |
| tel22h2-RGG (4905) replicate 1 | 100 |
| tel22h2-RGG (4905) replicate 2 | 100 |

**Table A2:** Failed attempts (Did not show instability of G-quadruplex)

| **Complex** | **Time (ns)** |
| --- | --- |
| TERRA12-RGG replicate 1 | 150 |
| TERRA12-RGG replicate 2 | 150 |
| TERRA12-RGG replicate 3 | 100 |
| TERRA24-RGG replicate 1 | 100 |
| TERRA24-RGG replicate 2 | 100 |
| TERRA24-RGG replicate 3 | 150 |
| TERRA24-RGG replicate 4 | 100 |
| TERRA24-RGG replicate 5 | 100 |
| TERRA24-RGG(205-219) replicate 1 | 50 |
| TERRA24-RGG(205-219) replicate 2 | 50 |
| TERRA24-RGG(205-219) replicate 3 | 100 |
| tel22h1-RGG | 100 |
| tel22h2-RGG truncated (218-227 AA) | 100 |
| tel22h2-GGRGG,YNGF motif (small functional motifs found from SLiM database | 100 ns each in 3 reruns |
| tel6-RGG replicate 1 | 100 ns |

The first hypothesis we arrived at in our study is the perturbation of GQ by turn-like structures of RGG stabilized by the stacking interaction of Phe with the G-tetrad. To validate our observations, we performed replicate simulations of several unfolders and one of the tel22h2 complexes show GQ perturbation in two different replicates. The RGG region interacting with the GQ in this complex adopts a turn-like structure stabilized by the stacking interaction of Phe (Fig. S6.1(a)). Fig. S6.1(b) and (c) show the H-bonds between RGG-GQ and the intra-quartet H-bonds indicating the perturbation of GQ. A similar turn-induced GQ perturbation was observed in the TERRA12 complex. From these complexes, we identified a fragment of RGG (205-219 AA) that showed a stable structure and interactions in this simulation. In order to test the ability of this fragment to stabilize or perturb the GQ, we simulated a complex of this truncated RGG with TERRA24. This fragment contains two FGG motifs and during the simulations, at the least one of the two Phe residues continue to interact with the GQ tetrad and induce the displacement of a K+ ion from the core (Fig S6.2(a)). Such perturbation of GQ structure by a truncated RGG is similar to the perturbation of TERRA12 GQ by the full RGG. We calculated the H-bonds between RGG and GQ (Fig. S6.2(b)), as well as intra-quartet H-bonds (Fig. S6.2(c)) in this simulation. From Fig. S6.2(c), it was clear that the GQ was stable initially, however, after ~70 ns of simulation, the number of intra-quartet H-bonds decreased. This is associated with a corresponding increase in H-bonds between RGG and GQ indicating RGG-induced changes in the GQ structure.


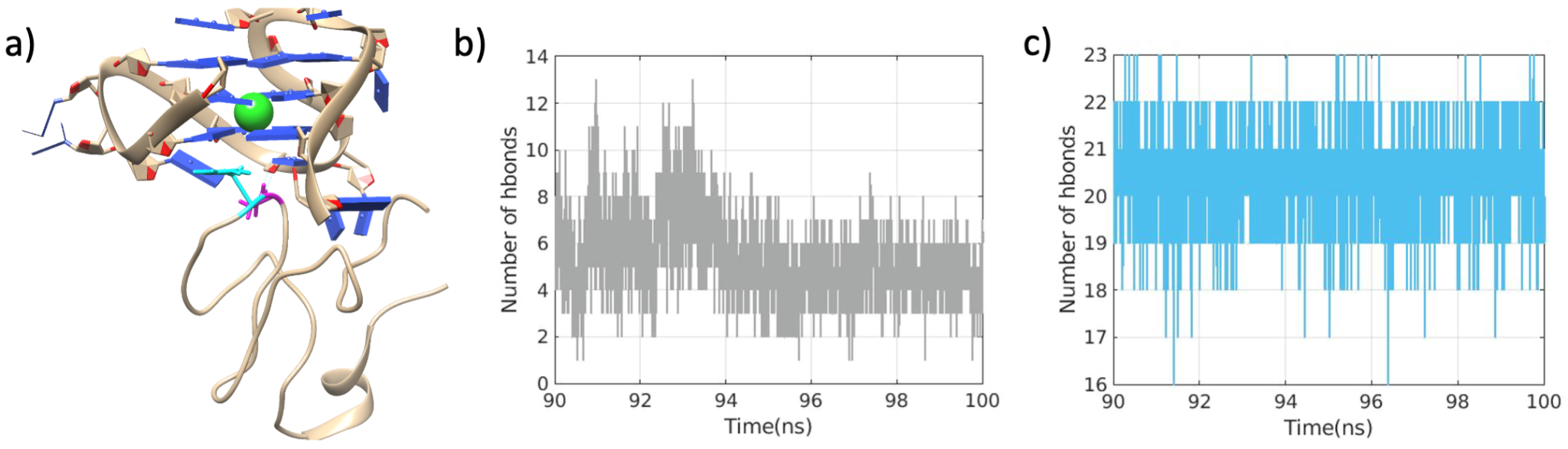
**Fig.S6.1:** a) tel22h2-RGG-box complex. Phe222 is marked in cyan. Asn221 is marked in magenta. Time evolution of hydrogen bond in tel22h2-RGG-box domain complex (100 ns simulation). b) tel22h2-RGG-box hydrogen bond. c) intra-quartet hydrogen bond.


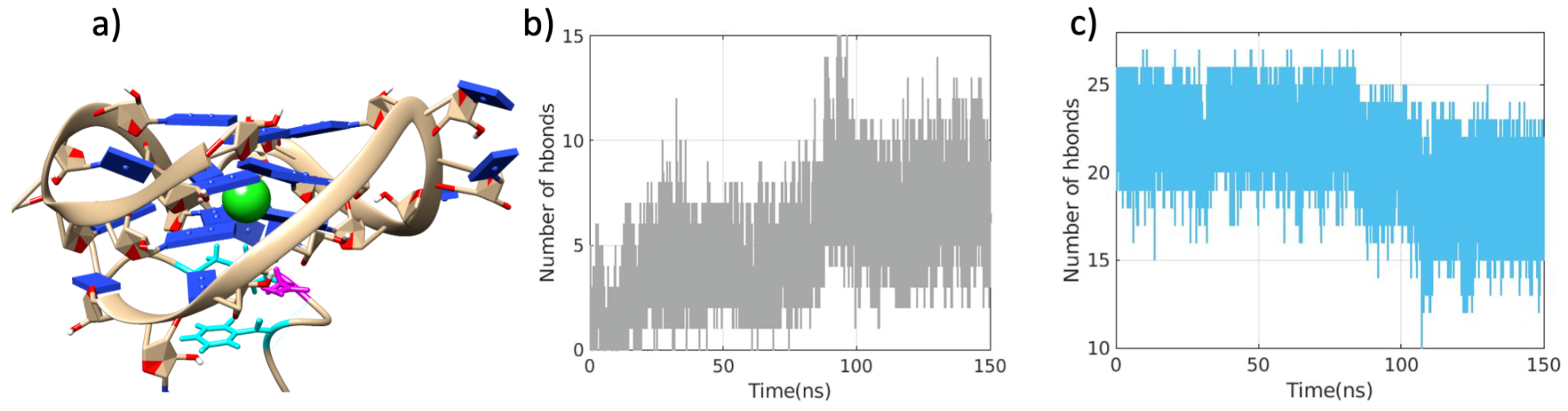
**Fig.S6.2:** tel24-RGG-box (205-219) complex. Phe202 and Phe208 are marked in cyan. Asn205 is marked in magenta. Time evolution of hydrogen bond in TERRA24-RGG-box domain (205-219) complex (150 ns simulation). b) TERRA24-RGG-box hydrogen bond. c) intra-quartet hydrogen bond.

The second hypothesis for the perturbation of GQ is via the grooves, due to the interaction of amino acids with multivalent and long sidechains. We observe such interaction in tel6 GQ, where the Asn249 expresses bifurcated H-bonds with an oxygen atom in the deoxyribose sugar of G5 and N3 atom in the Watson-crick face of G4. The simultaneous interactions with two adjacent strands of GQ leads to its unfolding. A similar perturbation is observed in a replicate simulation also. Hence, we decided to test the role of Asn or its multivalency on GQ stability by designing Ala and Arg mutants. Ala behaves as the negative control, while Arg is considered to be the positive control due to its ability to form multiple H-bonds. As expected, our simulations show that the N249A mutation does not perturb the GQ structure, while the N249R mutation induces instability. The H-bond plots of N249R mutation (Fig.S6.3(b) and (c)) clearly show the perturbation of GQ when the RGG interacts strongly. The loss of intra-quartet H-bonds in case of N249A mutation (Fig. S6.4(b) and (c)) is not RGG-induced, since the RGG loses interactions with the GQ during this period of instability. In one of the tel22h2 complexes, a similar interaction of Asn with the GQ grooves was observed and hence, we simulated this complex to check Asn-induced perturbations (Fig. S6.5). The Asn in this complex expresses a bifurcated hydrogen bond with T5 and G10 of adjacent strands (Fig. S6.5(a)). Upon simulating this complex, GQ perturbation is observed during the initial 5 ns and the GQ stability is rescued thereafter. The H-bond plots clearly show that a strong interaction of RGG with GQ induces perturbation in the GQ structure which is rescued upon the weakening of RGG-GQ interactions (Fig.S6.5(b) and (c)). A similar observation is seen in a majority of our simulations reinforcing the stochastic behavior of the complex that makes it difficult to capture by experimental techniques.

Altogether, instability in GQs is induced by either the β-turn structure, the coupled action of Phe-Asn, or, in some cases, a combination of both factors. All these evidences point to the direction that Phe by itself and the presence of Asn in the vicinity of the FG/FGG motif is one of the key contributors of GQ instability. We propose this as a testable hypothesis where further experiments are required.

**Fig.S6.3:**
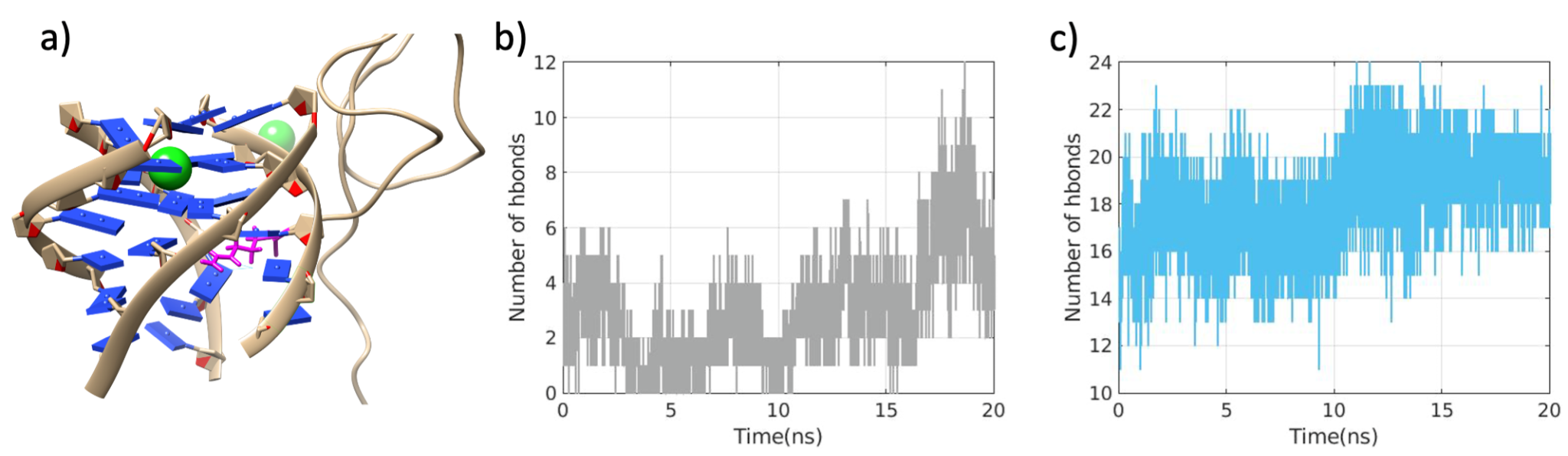
 a) tel6-RGG-box (Asn249Arg mutation) complex. Bifurcated hydrogen bond between Arg249, T8 is shown. Arg249 is highlighted with magenta color. Time evolution of hydrogen bond in tel6-RGG-box domain (Asn249Arg) complex (20 ns simulation). b) tel6-RGG-box hydrogen bond. c) intra-quartet hydrogen bond.


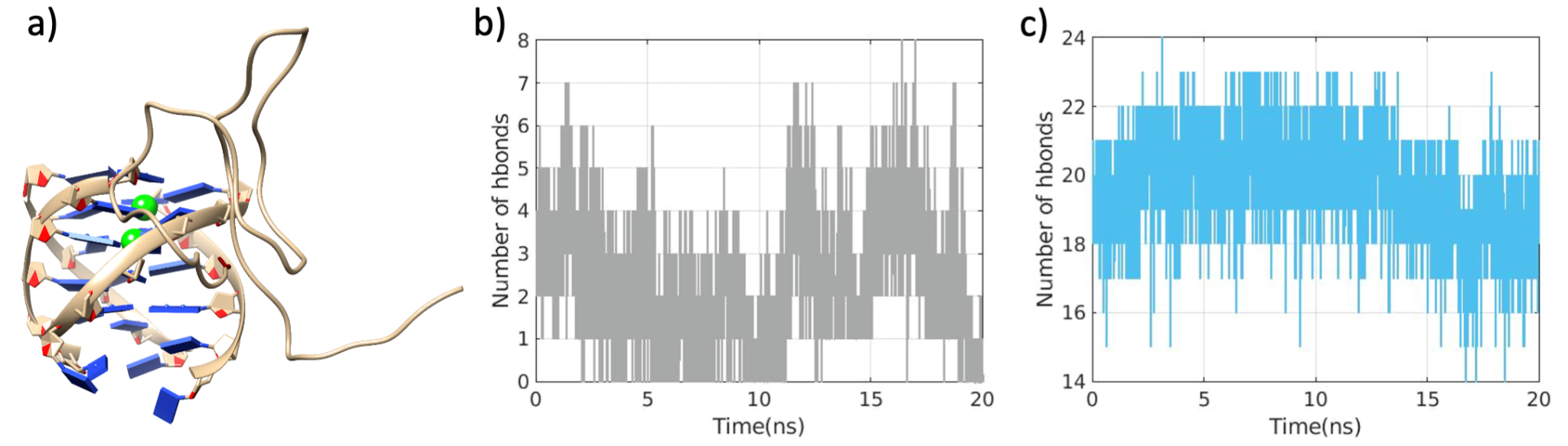


**Fig.S6.4:** a)tel6-RGG-box (Asn249Ala mutation) complex. Ala249 is highlighted with maroon color. Time evolution of hydrogen bond in tel6-RGG-box domain (Asn249Ala) complex (20 ns simulation). b) tel6-RGG-box hydrogen bond. c) intra-quartet hydrogen bond.


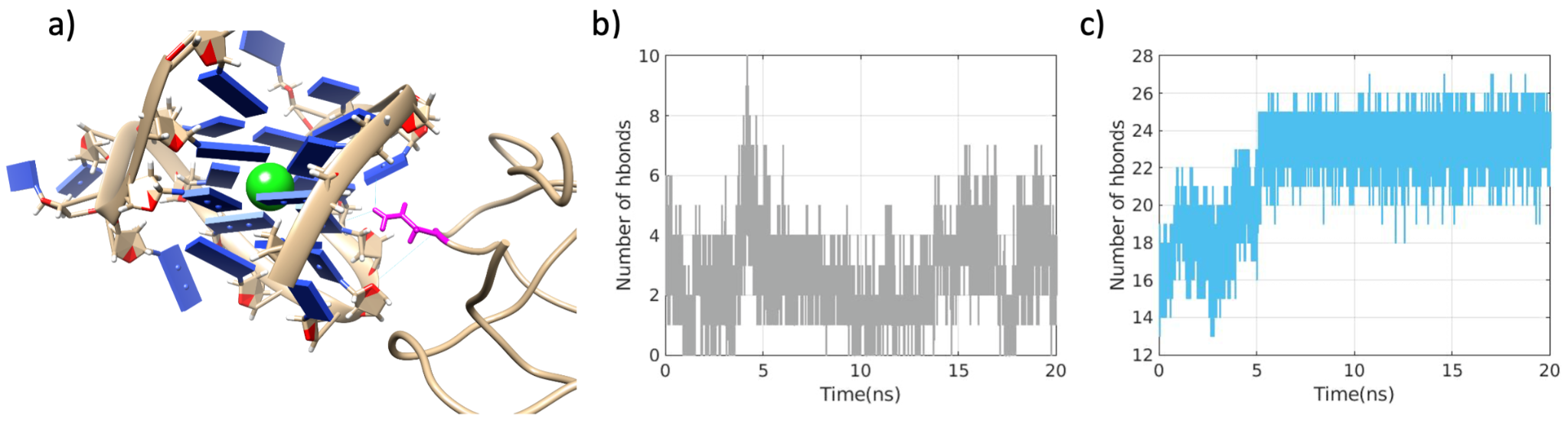


**Fig. S6.5:** a) tel22h2-RGG-box domain complex. Bifurcated hydrogen bond between Asn215, T5 and G10 is shown. Asn215 is highlighted with magenta color. Time evolution of hydrogen bond in tel22h2-RGG-box domain complex (20 ns simulation). b) tel22h2-RGG-box hydrogen bond. c) intra-quartet hydrogen bond.
